# Operational assessment of a border management mobile app for traveller pre-registration

**DOI:** 10.12688/openreseurope.22737.2

**Published:** 2026-07-28

**Authors:** Joanna Darmos, Ricardo Neisse, Sara Facchinetti, Enrique Cabello Pardos, Antonino Passarelli, Zdravko Kolev, Denis Destrebecq, Darek Saunders

**Affiliations:** 1 Research and Innovation Unit, Frontex – European Border and Coast Guard Agency, Warsaw, Poland; 2 Computer Science and Statistics Department, Rey Juan Carlos University, Madrid, Spain; 3 Italian Ministry of the Interior, Rome, Italy

**Keywords:** mobile app; border management; traveller pre-registration; operational pilot

## Abstract

This paper presents the results of the App4EES pilot project, which developed and assessed a mobile app solution for the pre-registration of biometric and biographic data to improve the border crossing efficiency under the Entry/Exit System (EES) in the European Union. The project involved a real operational testing of the app at Stockholm Arlanda Airport, with over 1,600 travellers successfully completing the enrolment steps and providing valuable feedback. The results show that the app is effective in improving the efficiency of the border crossing process, ensuring compliance with data protection and cybersecurity standards, and providing a positive user experience. The app was designed to be intuitive and user-friendly, allowing travellers to easily pre-register their biometric and biographic data, including facial images and travel documents.

To highlight the outcomes, this paper presents the first open and detailed evaluation of a traveller pre-registration system tested in real operational conditions with a high number of participants. The app demonstrated an average reduction of 30 seconds in border processing times for visa-exempt travellers, corresponding to a 25% improvement in overall efficiency compared to standard EES test procedures. Furthermore, the system effectively supported multi-traveller and family registrations, including minors, demonstrating its capacity to streamline pre-registration for diverse traveller profiles.

The project outcomes have significant implications for the development of future border management systems, as they demonstrate the potential of mobile app solutions to enhance border crossing efficiency, improve user experience, and ensure data protection and cybersecurity. The project results also highlight the feasibility of integrating a mobile app solution with existing border management systems, including the EES, as well as the need for close collaboration between stakeholders, including border authorities, airlines, and technology providers.

## Plain Language summary

In this paper the results of the App4EES research project are presented. The goal of this project was to develop and assess a mobile application aiming on increasing the efficiency of border checks considering the new biometric enrolment steps in the Entry/Exit System (EES) for third country nationals to enter in the European Union. The app was assessed at Stockholm Arlanda Airport, with over 1,600 travellers participating and providing valuable feedback. The results show that the app is effective in improving the efficiency of the border crossing process, ensuring compliance with data protection and cybersecurity standards, and providing a positive user experience. The app demonstrated an average reduction of 30 seconds in border processing times for visa-exempt travellers, corresponding to a 25% improvement in overall efficiency compared to standard EES test procedures. Moreover, the system effectively supported multi-traveller and family registrations, including minors, demonstrating its capacity to streamline pre-registration for diverse traveller profiles.

## 1. Introduction

According to the European Travel Commission (ETC), ^1^ Europe is expected to witness a significant surge in tourism in the coming years. International travel has been growing across the continent compared to previous years, with foreign arrivals of up to 12% year-on-year, based on data from European destinations. Compared to the pre-pandemic levels, the increase was about 6%. This growth is driven by the rising demand for travel, improvements in air connectivity, and increasing popularity of lesser-known destinations. In comparison, the number of travellers in Europe has been steadily increasing over the past decade, with a record 713 million arrivals in 2019. ^2^ However, the COVID-19 pandemic led to a significant decline in tourism in 2020, with arrivals plummeting to 458 million. Despite this setback, the industry has shown remarkable resilience, and the ETC forecasts a strong recovery, with travellers expected to surpass pre-pandemic levels by 2025.

The Entry/Exit System (EES) ^3^ is a large-scale information system developed by the European Union to modernize the management of external borders through the digital registration of entry and exit events of third-country nationals. The system pursues a dual objective. First, it aims to strengthen security by helping to detect irregular migration, identify overstayers, prevent identity fraud, and support law-enforcement activities. Second, it seeks to improve the efficiency and quality of border management processes by replacing manual passport stamping procedures with digital records, enabling increased automation, facilitating traveller processing, and supporting a more seamless border-crossing experience. Although the introduction of biometric enrolment requirements may initially increase processing times at border crossing points, solutions such as traveller pre-registration and self-service technologies have the potential to mitigate this impact while preserving the security benefits of the system.

The EES will require the collection and cross-checking of the traveller’s biometric data, including fingerprints and facial images, at external border crossing points. This change in the border control process is essential to ensure compliance with the Schengen Acquis, and will significantly impact the border check process. The implementation of the new process steps required by the EES is expected to increase the time spent per traveller at the border-crossing points [1], making the processing of travellers more difficult for both the traveller and the border guard. This impact, although expected to be prominent in the first months of operations, may decrease later as more travellers become returning visitors.

Border management is a multi-faceted and dynamic field that requires innovative solutions to tackle the challenges and risks posed by illegal migration, terrorism, smuggling, and cross-border crimes. Testing and validating these solutions are not simple processes, as they encompass technical, operational, legal, and social aspects, among others. ^4– 12^ The App4EES pilot project, initiated by Frontex, is the first large-scale project to develop and assess a mobile app solution for the pre-registration of biometric and biographic information to expedite the border-crossing process under the Entry/Exit System (EES) simulated conditions.

This study presents the first comprehensive and open distributed evaluation of a mobile application that enables full traveller pre-registration, integrating biographical data entry, facial biometric enrolment with liveness detection, electronic passport chip verification, and a condition-of-entry questionnaire within a single platform. Uniquely, the app supports both individual and group submissions, allowing families or organised groups, including minors, to pre-register jointly, thereby enhancing the efficiency of diverse traveller profiles. Designed for deployment across 27 EU Member States under the Entry/Exit System (EES) framework, the solution ensures compliance with EU data protection and cybersecurity standards. Importantly, the system was tested under real operational conditions and demonstrated excellent performance, usability, and interoperability, marking a significant advancement in digital border management innovation.

The project successfully demonstrated the potential of the proposed mobile app solution to enhance border-crossing efficiency, improve traveller experience, and ensure compliance with data protection and cybersecurity standards. The app, called “Quick Border,” was tested in a real operational setting at the Stockholm Arlanda Airport in Sweden, with over 1,600 travellers participating in the pilot. The results showed significant time savings for most travellers, with an average reduction of 30 s in border-crossing times for visa-exempt travellers. The project also highlighted the importance of clear communication, stakeholder engagement, and continuous improvement to ensure the success of the app. The outcomes of the App4EES pilot project provide a solid foundation for its broader implementation across Europe.

The key scientific and operational contributions of this work are summarised below:

• Presentation of the first large-scale operational assessment of a mobile application for traveller pre-registration under Entry/Exit System (EES) conditions, involving more than 1,600 travellers in a real airport environment.
• Development and validation of an integrated pre-registration solution combining biometric enrolment, liveness detection, electronic passport verification, and conditions-of-entry questionnaires within a single mobile platform.
• Design and validation of an interoperable architecture capable of supporting integration with multiple national border management systems and adaptable to different border crossing point types, including airports and maritime ports, facilitating future deployment across EU Member States.
• Introduction and assessment of a multi-traveller registration capability supporting families, minors, and organised travel groups.
• Demonstration of measurable operational benefits, including an average reduction of approximately 30 seconds in border processing times and a 25% improvement in efficiency compared with standard EES testing procedures.
• Design and validation of a privacy-preserving and cybersecurity-by-design architecture compliant with European legal and regulatory requirements.
• Provision of practical lessons learned and operational recommendations for future deployment of mobile pre-registration solutions across EU external borders.

The remainder of this paper is structured as follows: Section 2 introduces the state of the art. Section 3 provides details about the scenario and a detailed description of the approach followed to protect privacy and fundamental rights. In Section 4, the system design is presented and discussed. Section 5 describes the implementation and operational deployment of the proposed system. Section 6 presents and analyses the results obtained in the operational environment. Finally, Section 7 summarises the main lessons learned, the findings, and directions for future work.

## 2. State of the art

Mobile apps are currently used by many countries to enhance the border check process and ensure a high security level for their citizens. In this section, we provide an overview of existing mobile apps for **pre-authorisation** before travelling and pilot projects conducted with the objective of assessing voluntary **pre-enrolment** using Digital Travel Credentials (DTCs) ^13– 19^ in a similar way to the pre-enrolment mobile app described in this paper. Pre-authorisation apps are mandatory for all travellers fulfilling a certain condition, for example, in case a visa waiver programme is in place. Pre-enrolment apps are typically voluntary, and their goal is to speed up the border-crossing process because most of the required information to assist the decision made by the border guards is provided in advance. The app described in this paper is a voluntary pre-enrolment solution.

The apps for pre-authorisation are equivalent to the Electronic System for Travel Authorisation (ESTA) in the United States of America and the European Travel Information and Authorisation System (ETIAS), which will be implemented in the Schengen Area for visa exempt travellers in the near future. ETIAS, in contrast to ESTA and the other mobile apps described in this Section, will not include facial biometric verification or access to data from the electronic Machine-Readable Travel Document (eMRTD) chip found in passports compliant with the ICAO Doc 9303 standard. ^20^

Some of the apps currently available to travellers for pre-authorisation before travelling include **ArriveCAN** ^21^ from Canada, **Electronic Travel Authority (ETA)** ^22^ from Australia, **ESTA,** ^23^ and **Mobile Passport Control (MPC)** ^24^ from the United States, and Korea **Electronic Travel Authorisation (K-ETA)** ^25^ from South Korea. The goal of these apps, except for MPC, which is a pre-enrolment app, is to pre-authorise visa exempt travellers before their trips and is a pre-requisite for travellers since they most likely will not be authorised to enter the country or board a plane to the destination without a confirmed pre-authorisation issued by the apps.

**ArriveCAN** is a mobile web-based app developed by the Canadian government to facilitate the arrival process in Canada for travellers. It was first introduced in 2020 in response to the COVID-19 pandemic to help track and manage the spread of the virus and other public health risks. The primary purpose of ArriveCAN is to provide travellers with a convenient way to submit their travel information and health declarations electronically to facilitate a more efficient and streamlined arrival process. Using the mobile app, travellers can submit: (1) their travel information, including flight details, accommodation, and contact information; (2) their health declaration, including questions about their vaccination status, symptoms, and potential exposure to the virus; and (3) their quarantine planning, including where they will stay and how they will access essential services. After submission, travellers receive arrival notifications and reminders regarding their arrival and quarantine requirements. ArriveCAN is mandatory for all Canadian and foreign national travellers, with or without permanent residence in the country. All data submitted by using the app using a mobile app or web-based version must be self-certified as truthful information, as false declarations can result in fines or other penalties.

The **Australian Electronic Travel Authority (ETA)** is a mobile app available for both iOS and Android devices that allows eligible travellers to apply for and manage their Australian ETA on their mobile devices. The app allows for application to the ETA, including tracking the status of digital access and storage. To apply, travellers must upload travel documents, such as your passport and proof of onward travel. The app also performs facial biometric verification to verify the traveller’s identity and additional modules to receive notifications and reminders about the ETA application status, travel requirements, and help and support, including access to FAQs, contact support, and request assistance with the ETA application.

**Mobile Passport Control (MPC)** is a mobile app that is available for both iOS and Android devices provided by the U.S. Customs and Border Protection (CBP) authority. It allows eligible travellers to submit their customs declaration (CPB Form 6059 B), passport, and VISA information through their mobile device before arriving in the United States of America. Eligible travellers include U.S. and Canadian citizens with a valid passport, as well as lawful permanent residents of the country. The app performs biometric facial verification and provides an electronic receipt after the data are submitted, including a QR code that serves as proof of completion. During the border check process facilitated by app users in over 30 U.S. airports, users of the mobile app can skip traditional customs lines and proceed to dedicated processing lanes.

**Korea Electronic Travel Authorisation (K-ETA)** is an electronic travel authorisation system introduced by the Korean government to simplify the entry process for eligible foreign nationals. The goal is to simplify the process of boosting tourism to the country. The K-ETA is designed to streamline the application for pre-authorisation of entry for eligible VISA exempt and waiver foreign nationals. The K-ETA provides an additional layer of security by checking the applicants’ backgrounds and travel histories. The K-ETA is accepted for selected airports and ports of entry in Korea. Eligible travellers can submit an online application by filling out the online application form on the K-ETA website, paying the required application fee, and uploading documents including a canned copy of their valid passport (at least six months of validity), a recent photo, and other required documents including return/onward ticks and proof of sufficient funds for their stay. After submitting the application form, travellers must wait for the processing and subsequent approval or rejection of their application, which usually takes place within 24 h of submission.

The **DTC1 Pilot** ^26^ in the Netherlands, **Aruba Happy One Pass** ^27^ for Aruba, and **Known Traveller Digital Identity (KTDI)** ^28^ were jointly supported by Canada and the Netherlands.

The DTC1 pilot in the Netherlands is a trial programme launched by the Dutch government in collaboration with the KLM airline to test during 2022 and 2023 the feasibility and benefits of using Digital Travel Credentials (DTCs) to facilitate border checks for travellers arriving at Schiphol Airport. In the DTC1 pilot, DTC type 1 (DTC1), according to the ICAO specification, ^13^ is generated and stored in the traveller’s mobile device. DTC1 is a digital token that contains a traveller’s biographic and biometric data (facial image).

The **Aruba Happy One Pass** is also a DTC type 1 solution launched in 2020 by the government of Aruba, aiming for a seamless and hassle-free travel experience for visitors. The pilot programme was developed in collaboration with the Aruba Tourism Authority, Queen Beatrix International Airport, and several airlines. The programme is currently available to travellers from the United States, Canada, and several European countries and is expected to expand to other countries in the future.

**Known Traveller Digital Identity (KTDI)** is a digital identity system designed to enhance the security, efficiency, and convenience of international travel using advanced biometric and artificial intelligence technologies to verify the identity of travellers and provide a secure, seamless, and personalised travel experience. KTDI has been piloted in several countries, including the United States, Canada, Germany, and the Netherlands.

Most of the existing apps, including the app described in this paper, implement a common process including self-declaration of the traveller information and journey, upload of supporting documents and completion of entry questionnaires, reading and validation of the eMRTD chip information (biographic and facial image data), and biometric verification with some degree of liveness assurance verification. The main goal of these apps is to increase security and improve the efficiency of the border check process, reduce waiting times, and improve travel experience.

Table 1 below provides a comparative analysis of the main characteristics, as they are publicly available, of the applications reviewed in this section and the Quick Border App.

**Table 1.** Comparative assessment of analysed applications.

Solution	Travel Authorisation	Facial Biometrics	Liveness Detection	eMRTD Chip Reading	Group Registration	Real Operational Testing	Integration with Border Systems
ArriveCAN	✓	✗	✗	✗	✗	✓	Limited
ETA Australia	✓	✓	Partial	✗	✗	✓	Limited
MPC	✗	✓	✓	✗	✗	✓	✓
DTC1 Pilot	✗	✓	✓	✓	✗	Pilot	Partial
KTDI	✗	✓	✓	✓	✗	Pilot	Partial
QuickBorder (proposed)	✗ (pre-registration)	✓	✓	✓	✓	✓ (1,600+ travellers)	Intended for all Schengen countries

While several existing traveller applications focus primarily on travel authorisation, customs declarations, or isolated digital identity functionalities, the proposed QuickBorder solution combines biometric enrolment, liveness detection, electronic passport validation, conditions-of-entry questionnaires, and multi-traveller registration within a single operational platform.

Unlike previous initiatives that were either limited to pilot demonstrations or specific digital travel credential concepts, QuickBorder was evaluated under real operational conditions at a major international airport and integrated with national border management systems. QuickBorder was designed as an interoperable pre-registration solution capable of integrating with different national border management systems and supporting multiple types of border crossing points, including airports and maritime ports, as well as use cases including both entry and exit procedures.

Unlike most existing traveller applications, which are typically developed to support the operational and legal requirements of a specific country, the proposed solution was conceived to accommodate the heterogeneity of border management infrastructures and procedures across EU Member States, thereby facilitating future large-scale deployment within the EES framework. Furthermore, the solution was designed from the outset following privacy-by-design, cybersecurity-by-design, and regulatory compliance principles, providing a comprehensive framework for future EES-compatible traveller pre-registration systems for the Schengen Area.

## 3. Project description

The App4EES pilot project developing the QuickBorder App was implemented by the Research and Innovation Unit (INNOVATE) of Frontex, upon request by the European Commission. The project was implemented from October 2023 to September 2024, with six weeks of testing in a real operational environment in May and June 2024.

The scope of this project was to develop and assess a mobile solution for the pre-registration of travellers’ information, including their biographic and biometric data (facial images) before their arrival at a border crossing point (BCP), giving access to the Schengen Area. The project objectives were as follows:

• **Operational viability**: An in-depth examination of the viability of a secure and usable mobile pre-registration app through an operational trial and a retrospective analysis of the desired features and applications in view of a future European solution.
• **Pre-registration effectiveness**: Assess the effectiveness and efficiency gains of streamlining the border-crossing process by pre-registration of eMRTD, facial image, and conditions of entry questionnaire data.
• **Border check process efficiency**: Test the integration of the app in the national border management system to see how the app can allow an MS to gain time when collecting, verifying, and using traveller data in advance.
• **User experience**: Insights into passengers and border guards’ perceptions and expectations related to a pre-registration solution collecting biometric data.
• **Compliance assessment**: Perform a thorough assessment and produce documentation aligning the solutions to data protection and fundamental rights requirements.
• **Process assessment**: Develop and assess an EES process flow for an air BCP that includes the app as a self-service system.

### 3.1 Methodology framework

The methodology followed in the App4EES project is based on the Frontex methodology for pilot project development. Pilot projects are essentially limited-scale tests in real operational situations to evaluate the feasibility and outcomes of a selected technology. Under this general scheme, the approach follows a set of defined steps.

The first was devoted to obtaining a functional app that could be used by travellers to send data in advance to the border control point. Once a testing version of the app was developed, an initial test was performed to evaluate its functionality and user interface with real travellers. This test was conducted at the Schiphol Airport in The Netherlands, and no data were transmitted to any system. This initial test was used to adapt and tailor the interface and to facilitate user navigation in the app.

The second test was conducted in Arlanda Airport, Sweden and involved real travellers, whose data was sent to the authorities to facilitate border crossing.

The global objectives of the project were grouped into three categories to be assessed by a set of defined Key Performance Parameters that could be collected to evaluate the performance and effectiveness of various processes within the operational phase of the pilot project.

The macro categories ensured alignment between the indicators and the objectives of the App4EES operational pilot. They covered the areas of expected impact of the application when integrated into the national border crossing process on both EES-related functionality and the conditions of the entry questionnaire. The authors considered three macro categories:

• **Category 1 - Mobile App & Backend:** This concerns the developed QuickBorder application and the backend designated for communicating with the BCP. It covers indicators that focus on the effectiveness and usefulness of the application and the process it has towards the back-end.
• **Category 2 - BCP Integration:** This concerns the integration of QuickBorder with the border crossing point infrastructure. The indicators focus on the effectiveness of the integration mechanisms in the BCP.
• **Category 3 - Business Process:** Concerning the impacts on the overarching business processes during border crossings in which QuickBorder is used. The indicators focus on the usefulness and impact on business processes improved by the App.

### 3.2 Key performance indicators description

To establish a robust and multi-faceted evaluation framework for assessing the attainment of predefined objectives, a specific Key Performance Indicator (KPI) was deliberately selected, thereby enabling a quantitative and objective assessment of progress and success. The accompanying table defines the Key Performance Indicators (KPIs) and facilitates a clear line of traceability with the categories, thereby enabling a systematic and transparent evaluation framework.

**Coverage of travel documents/countries**: Coverage of travel documents/countries according to the documentation of the chosen solution.

**Total number of registrations and identification of the ending point of the registration:** How many transactions were completed, and identification of the stage of process in which the user finished the enrolment on the app. The reasons for process abortion were as follows: travel document could not be captured, which could be related to OCR errors; face could not be captured during liveness detection; live captured image and/or the eMRTD image were not successfully captured, which related to reading the image from the chip; liveness detection was negative; live captured image and the eMRTD image were not successfully matched.

**Total number of users reached with campaign for app piloting:** How many users were contacted to participate in the pilot.

**Total number of downloads of the App in both stores:** How often was the app downloaded from the Play Store and App Store?

**Process step at which registration was aborted by user or by the system:** Process steps at which a registration/journey was permanently aborted by the user or the system. An aggregated number should be provided for each process step.

**Reason of abortion of registration by user:** Feedback asked by the user if they manually aborted the registration.

**Total number and percentage of completed and aborted registrations:** Completion and rate of the full registration process.

**Percentage of app type (iOS/Android) used in completed registrations:** Ratio of iOS/Android users in completed registrations.

**User satisfaction survey (app users and border guards):** Survey presented in a paper/electronic form to pilot participants and border guards.

## 4. System design

This section describes the architectural design of the QuickBorder mobile app, including frontend and backend services. After introducing these architectural elements, cybersecurity, data protection issues, and implemented countermeasures were discussed.

### 4.1 Architecture description

**Figure 1.**
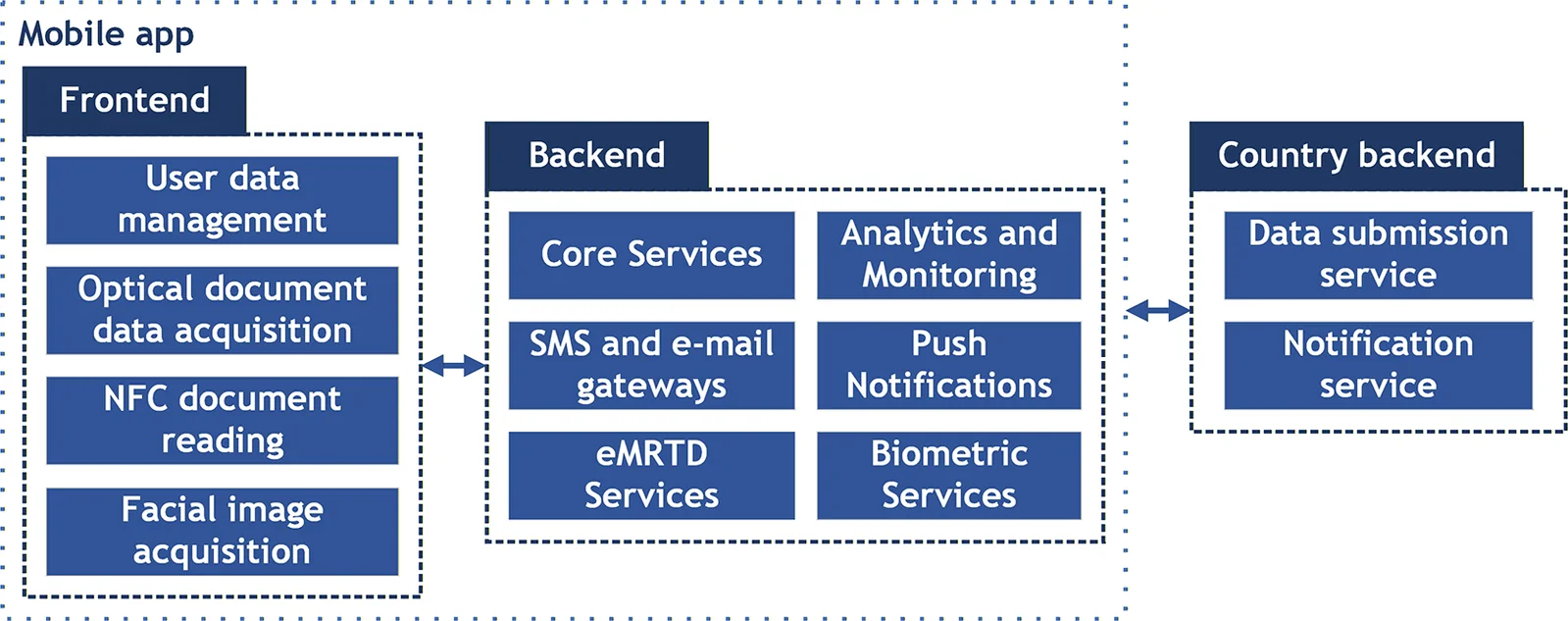
Mobile app high level architecture.

The design of the mobile app architecture (see Figure 1) is divided into three main parts: the app frontend, app backend, and country backend. The frontend is operation system-specific (iOS or Android), runs exclusively on the user’s mobile device, and includes services, libraries, and Software Development Kits (SDKs). These support the management of user data related to travel documents and journeys, optical document data acquisition, near-field communication (NFC) document reading, and facial image acquisition. The backend includes core services, analytics and monitoring, gateways to enable Short Message Service (SMS) and e-mail validation, push notifications, electronic Machine-Readable Travel Document (eMRTD) services, and biometric services. The country backend implements services to receive data submissions from travellers and a notification service that sends confirmations or additional journey notifications to the user’s mobile device.

From a functional perspective, the mobile app frontend implements only the minimal functionality necessary, including secure user data management, and relies on the backend for the implementation of core services and other related functionalities. The architecture of the mobile app addresses two main issues related to security and data assurance: (1) the end-user mobile devices running the frontend do not communicate directly with the country backend and (2) any data assurance or security-sensitive operation related to eMRTD or biometrics is performed within a trusted backend service.

The following subsections detail the front-end functionality and user interface, the backend services implemented, and the data protection and cybersecurity concerns and countermeasures implemented.

### 4.2 Frontend user interface

The front-end consists of native mobile apps implemented for **iOS** (version 15+) and **Android** (platform 8.0 Oreo API level 26) mobile devices with near-field communication (NFC) capability. The app was built using native frameworks relying on the Kotlin Multi-Platform to cover end-to-end functions for the traveller to pre-register data before travelling to/from the Schengen area.

The mobile app incorporates embedded Software Development Kits (SDKs) and consumes the following backend Application Programming Interface (API) services:

• NFC communications.
• eMRTD OCR data scanning.
• Configuration via Rest APIs provided by Mobile Backend service.
• Liveness Assurance capturing.
• System logging via Rest APIs with the Dynatrace tool.

**Figure 2.**
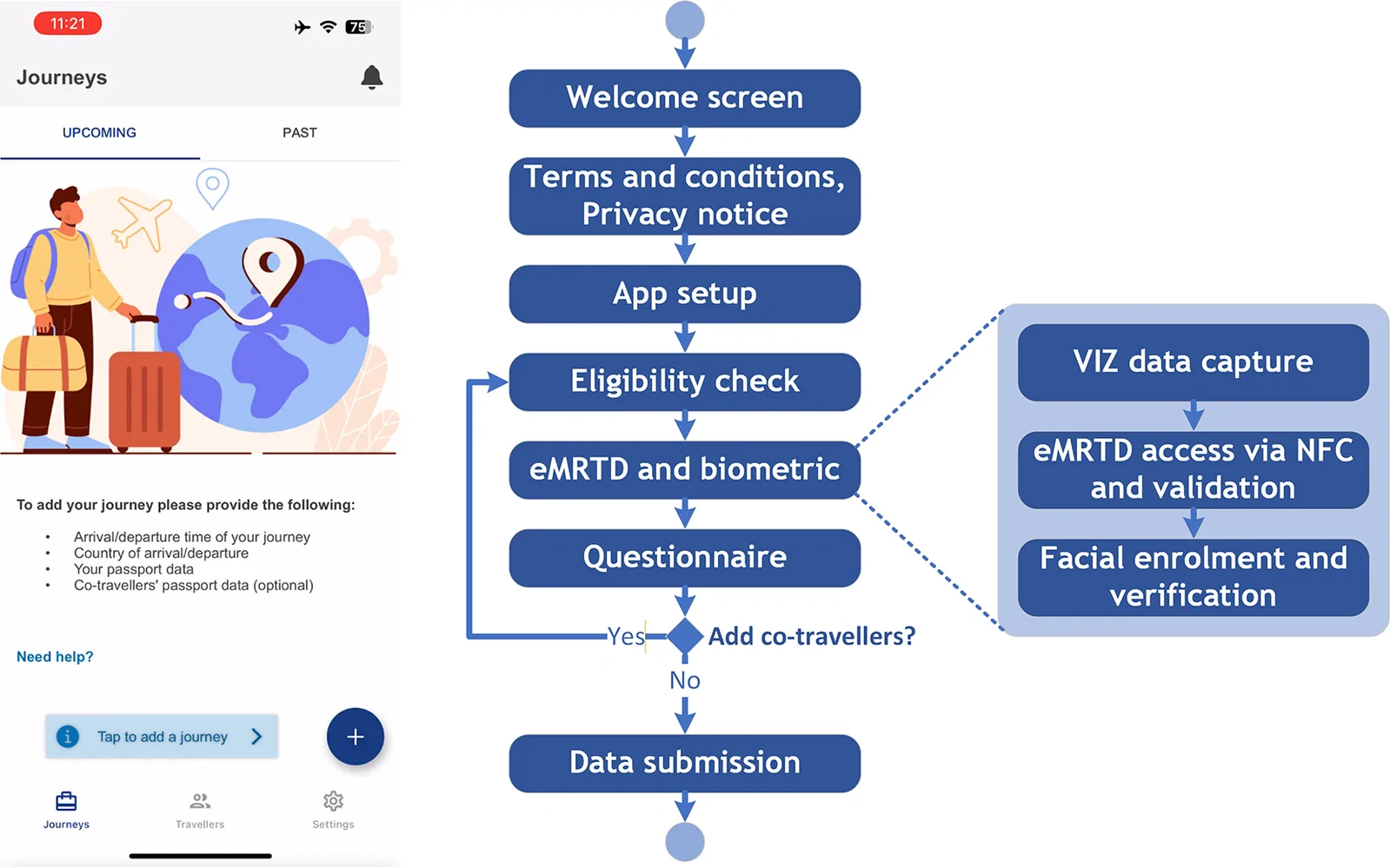
App frontend interface to create journeys and traveller process flow.

The app frontend (see Figure 2) enables the traveller to perform the following operations:

1. **Welcome screen**: Display general information about the app.
2. **Terms and conditions, Privacy Notice**: Travellers are informed about the terms, conditions, and privacy notice.
3. **App setup**: The traveller sets up an access PIN and optionally configures a biometric login if supported by the mobile device and user interface language (three languages were supported during the operational pilot: English, Spanish, and French).
4. **Eligibility check**: The traveller is asked about nationality and residence permit status to ensure that only travellers that should be enrolled by the EES are pre-registered using the mobile app.
5. **eMRTD and biometric:** It consists of the following three sub-steps.
  a. **VIZ data capture**: The mobile app reads the Machine Readable Zone (MRZ) and the biographic data page of the travel document using optical character recognition (OCR).
  b. **eMRTD access via NFC and validation**: The eMRTD chip from the travel document is access/read; clone detection is performed if supported by the chip, and digital signatures are performed.
  c. **Facial enrolment and verification:** The traveller facial image is enrolled and the identity is verified, including liveness detection and biometric face verification against the travel document. Liveness detection is performed to detect presentation attacks using a mechanism implemented with flashing colours and a live video feed sent to the app backend.
6. **Questionnaire:** The traveller is asked to answer some questions configured by the border crossing point regarding certain conditions of entry/exit to/from the Schengen area.
7. **Add co-travellers**: For each journey, the traveller can add co-travellers (adults or minors) who have to go through the identity verification process and to fill in the conditions of entry/exit.
8. Full **pre-registered data submission** to the National Backend system via mobile app backend services: The traveller can submit the pre-registered journey data to the National backend of departing/arriving border crossing point in the Schengen area or use the generated QR code to reconcile data at the border (according to the border crossing point configurations in the mobile app backend).

The traveller is also able to configure the app localisation and settings. Concerning localisation, the app automatically detects the phone language at the start and, if different from the app localisation languages, it is automatically set on the default language interface (English language). Moreover, the traveller can configure the preferred login method (PIN or phone biometric).

The mobile app depends on device compatibility, networking, keyboards, cameras, NFC readers, and other supporting services. Facial biometric enrolment is performed using the traveller’s native smartphone camera without requiring any dedicated external sensor. Modern smartphone cameras provide image quality that is generally sufficient for biometric verification purposes, particularly when combined with the implemented quality assessment and liveness detection mechanisms. The objective of the mobile enrolment process is to perform remote identity verification and facilitate pre-registration before arrival at the border. However, if the image acquired through the mobile device does not meet the quality thresholds required for enrolment in operational EES records, a compliant facial image can still be acquired at the border crossing point using border-control equipment. Consequently, the mobile application complements rather than replaces existing border biometric acquisition procedures and provides operational flexibility while maintaining compliance with applicable biometric quality requirements.

The app user interface enables interaction with the traveller to collect user authorisation and agreement to share data, collect the required pre-registration data, and present third-party requests to create verifiable presentations from one or more attestations. The app user interface keeps the traveller continuously informed and requests confirmation of the user’s authorisation before exchanging or presenting any data.

The mobile app layered architecture is composed of the following components:

• UI: Native composition for Android/native SwiftUI for iOS, UIKit for native iOS development, Android XML for traditional Android UI
• Presentation layer: view models and navigation through Kotlin Multiplatform Mobile technology (KMM)
• Business logic: Through KMM Interactors, use cases, domain models, state management, and validation
• Data layer: Repositories and Data Transfer Objects (DTOs)

KMM and Native UI interact based on the Model View Interactor (MVI).

The following technologies are utilised for mobile app development.

• Android: Kotlin 1.8+, Jetpack Suite, MVI Architecture, Gradle, Firebase Suite (Crashlytics, FCM), Hilt-Dependency Injection, Junit, Mockito, Turbine, Fastlane (Automation)
• iOS: Swift 5+, SwiftUI, MVI Architecture, Swift Package Manager, Combine, Firebase Suite (Crashlytics, FCM), XCTests, SwiftLint, Mockingbird or SwiftyMocky, Fastlane (Automation)

The mobile app user interface accessibility was designed and evaluated based on the WCAG 2.1 standard in line with the Harmonised European Accessibility standard EN301 549 V3.2.1 (accessible text and images, keyboard navigation, colours and contrast, responsible design, accessible links and buttons, media accessibility, ARIA – Accessible Rich Internet Applications – and attributes, focus management).

The mobile app visual identity is based on the EU commission eUI-UX guidance and eUI mobile components (Figma community), and clearly identifies the context of a Frontex pilot project. Travellers who took part in the pilot were provided with an activation code to be used at the first use of the app as authorisation to access the app’s main functionalities. This verification step ensured that only authorised travellers could access and use the app’s features.

The app includes welcome screens to provide essential information about the goals of the app and its features, with the possibility of skipping guidance information. Before starting to use the app, the traveller is required to accept the terms and policies of the app to proceed further. To secure the data, the traveller is required to create a login PIN with the possibility of enabling a biometric login (if such a service is enabled by the user’s mobile phone) after successful PIN creation.

The traveller creates a journey selecting the travel date, departure/arrival country, and border crossing point and performs identity verification (document scan, NFC, liveness detection, and biometric verification). The traveller is asked to fill in the conditions of entry/exit, and the pre-registered data are ready for submission to the national backend system. It is possible to add a co-traveller to the same journey. When the submission is completed, a notification (push notification and in-app notification) is received by the traveller regarding the status of the journey (e.g., accepted or rejected) and other useful information for travel. A QR code for each traveller is available to reconcile the data at the border crossing point when needed. The passport data and selfie acquired during the liveness detection process have an expiration date according to the applicable regulations and specific security needs.

From the app settings, the traveller can change the app language, export local data for safekeeping in a chosen file, delete all data if no longer needed, change the PIN and/or enable biometric login, view the terms and conditions (if any change occurs, the traveller will be requested to approve it again at the following login), get started (welcome screens) and how to use the app sections, frequently asked questions (FAQs), an option to report an issue to the app owners, an option to rate the app including the sharing of experiences and rating in the app stores, information about the app current version that includes the version number and the environment (test or production), and a log out to ensure security.

### 4.3 Backend services

**Figure 3.**
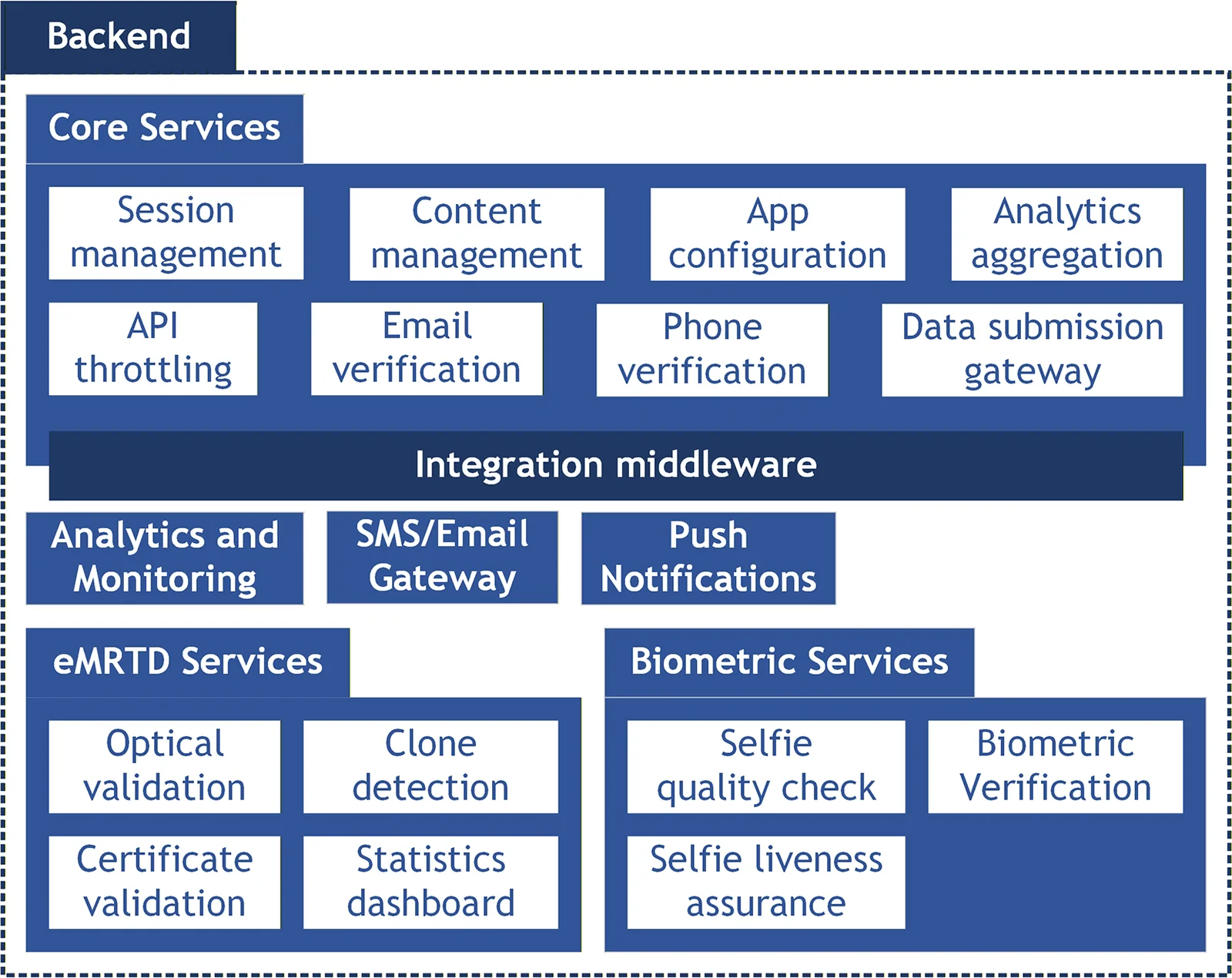
Implemented services.

The diagram in Figure 3 details the services implemented by the mobile app backend, organised according to their functional goals. These services are orchestrated using integration middleware and are common to both Android and iOS versions of the app. The following service modules were implemented.

• **Core services** are responsible for the management of user sessions, Application Programming Interface (API) throttling, content management in multiple languages, configuration of the app and country parameters, verification of user phones and e-mail, aggregation of analytics across multiple service modules, and data submission gateways.
• **Analytics and monitoring:** All service modules were used to record anonymised statistics related to app functionality usage and track performance.
• **SMS/Email gateway**: This provides a gateway interface to send text (SMS) and e-mail messages to support the validation of registered phone numbers and e-mail addresses and communicate with travellers.
• **eMRTD services**: Provides services to validate optical features of travel documents, detect clones, validate country signing certificate authorities (CSCA) using a reference masterlist, and a dashboard of statistics related to travel documents including country of origin, document issuer, data of issuance, and document features.
• **Biometric services**: The liveness assurance process of the facial image (selfie) implements the quality check of the selfie enrolled using the frontend and performs biometric verification of the selfie with the image retrieved from the chip of the travel document using NFC.
• **Push notifications**: Implement push notification functionality to enable the country to send additional information to end users.

## 5. Implementation and operational deployment

This section describes the technological implementation details of the app frontend and backend, including the integration aspects of the app with the country backend infrastructure and the subsequent use of the pre-enrolled data in the border crossing points.

### 5.1 Technical implementation

The mobile app was developed for both Android and iOS devices using Kotlin, a framework in which common code is shared for both mobile operating systems. As introduced in Subsection 4.3, robust backend services were implemented because the user’s device cannot be considered a trusted environment. Therefore, controls must be put in place to ensure that the app is running on a real device (not a simulated or virtual machine) and that the mobile device’s security has not been compromised, for example, by using jailbreaking tools. Mobile app backend services run in a cloud infrastructure using a virtualised platform for services, which also implements controls to protect against flooding and abuse from end-user devices, which may be under the control of potential attackers.

### 5.2 Integration with national backend systems

The Swedish National Authority accepted its integration with the QuickBorder pilot application and performed on-site operational testing of the app at Stockholm Arlanda Airport. The integration of the QuickBorder process in the Member States’ border checks workflow allowed the simulation, to a certain extent, of the processing of travellers under the conditions of the EES operation. The two contributing countries, the Netherlands, Sweden, and Frontex, considered different ways of making journey submissions available to national authorities. The solution that was agreed on prioritizes the fastest possible expedition of the journey submission to the country backend, minimizing the steps, and avoiding the complexity of having the app communicate with more than one backend. The solution of choice is a central mobile app back-end that supports the app and receives the submissions, but then immediately passes these submissions to the country’s submission service.

When a journey is submitted to the Swedish Submission Service, it is stored in a separate location awaiting reconciliation during the border check. Initially, it was planned to add each journey as a new border crossing in the border application, but since the traveller may submit several journeys as long as they are within the limits of a set period prior to travel, the border application would not know which one to reconcile upon first crossing. By storing the submissions in a separate location, the border application can pick up the submission that matches the expected arrival best from the submissions database.

When the traveller reaches border control, the pre-registered information must be fetched from the submission database. The fastest option available is to use the face image as a token and take verification photos on the fly when the traveller approaches the border booth. These photos were matched against the recorded pre-registrations. Together with DTC-style reversed verification, the pre-registered submission can be fetched by face image, and the travel document can be verified with a tap on an NFC reader. During the pilot operational testing, however, reconciliation was performed by scanning the traveller’s passport. When the travel document was read, the pre-registered information was fetched from the submissions database, automatically filling the preassigned fields in the border guards’ first-line application. App integration was performed for all booths in Arlanda, allowing many on-duty guards to trial the new process.

The operational tests were conducted in parallel with the technical testing of cameras and fingerprint readers set up in anticipation of EES entry into operations. This allowed for the timing of the entire process and a comparison of the processing times with and without the mobile app.

## 6. Privacy and data protection, fundamental rights, and cybersecurity

This section describes privacy and data protection, fundamental rights, and cybersecurity considerations during the design and operationalisation of the mobile app implemented during the pilot project.

### 6.1 Privacy and data protection

The collection and processing of biometric and biographic information within a mobile environment introduces significant privacy and data protection challenges. The QuickBorder solution was designed to operate in real operational environments using real traveller data, making the protection of individuals’ personal information a primary design objective. The implemented privacy-by-design and privacy-by-default approach addresses risks associated with excessive data collection, unauthorised access, unlawful processing, and lack of transparency. Data minimisation mechanisms ensure that only information strictly necessary for the pilot objectives is collected. Explicit informed consent, transparent privacy notices, clearly defined retention periods, and mechanisms allowing travellers to revoke consent and request deletion of their data ensure compliance with the principles established by the General Data Protection Regulation (GDPR). In addition, a dedicated Data Protection Impact Assessment (DPIA) was conducted to identify, assess, and mitigate risks associated with the processing of biometric information, ensuring full accountability, transparency, and alignment with European legal and regulatory requirements.

Furthermore, despite the fact that the AI Act was not yet applicable during the execution of the pilot, the DPIA incorporated an assessment of risks and mitigation measures aligned with the provisions under discussion in the draft AI Act. This proactive approach facilitated early alignment with anticipated European requirements for AI systems involving biometric processing and strengthened the overall accountability and future readiness of the solution.

Ensuring robust data protection and compliance with data privacy laws was the cornerstone of the App4EES pilot project. The general design principles were based on *privacy by design* and *privacy by default* approaches. The legal basis for participation in the pilot testing activities, including the provision of data by travellers, was conducted on an entirely voluntary basis. Since the EES was not yet in operation at the time of the pilot project, participation relied on explicit consent, which was well aligned with the voluntary nature of the provision of data.

The initial step in the project data protection strategy involved a thorough analysis of the roles of the main actors involved in processing the personal data of the travellers. After discussions with the Member State representatives, it was concluded that the separate controller setup between Frontex and the hosting Member State would be viable. This foundational step ensured that all parties were aware of their roles and could implement appropriate measures to safeguard their personal data.

Following the role analysis, key privacy-related documentation, such as privacy notices, consent clauses, and authorisations, were drafted and provided to travellers. These documents were crafted with the aim of being clear, transparent, and informative, thereby ensuring that travellers were fully informed about how their data would be used and protected.

One of the features developed in the app allows one journey to be used to collect personal data from a group of travellers and send the data in one package. If the traveller wanted to add personal data from a co-traveller, all the requirements and steps to be performed were clearly detailed in the app to ensure that informed consent from the co-traveller was obtained. If the co-traveller is a minor, and to provide special protection to the minor’s personal data, a special and separate requirement option has to be accepted, which enhances data protection. Only legal guardians with unlimited parental authority or duly authorised individuals can provide personal data from minors.

A critical component of data protection efforts is the Data Protection Impact Assessment (DPIA). DPIA was conducted to identify and mitigate any potential risks associated with the processing of travellers’ data. This assessment involved a detailed examination of the data flows, processing activities, data subjects, data categories, applied technologies, and potential vulnerabilities. By identifying risks, the project team implemented robust safeguards and controls to protect personal data from unauthorised access, breaches, and other security threats. This assessment was performed in close cooperation with the Frontex Data Protection Office, setting up a specific team responsible for this task. Even though the AI Act and regulations were not in force during the operationalisation of the app, DPIA explored the risks and mitigation measures related to the use of AI in the app.

From a data protection point of view, the implemented strategies included:

• **Data minimisation**: An eligibility check must be performed before collecting personal data. Personal data were collected only from travellers who met these requirements. From these travellers, only the data needed to fulfil the project objectives were collected.
• **Traveller information**: All pilot participants were informed of how their data would be used and that they would have the possibility to choose not to participate in the pilot, without adverse effects. Only after the travellers accepted the privacy notice and the terms and conditions could personal data acquisition start. Informed consent was obtained from all volunteer participants of the project.

Data on the mobile phone were under the control of the traveller, and a retention period was set to keep data on the backed only for the minimum time required. Consent could be revoked at any time, and data were removed. The data were kept separate, and no fusion of biometric and biographical data was allowed for backend processing.

### 6.2 Fundamental rights

The Fundamental Rights Office was consulted not only to provide safeguards, but also to promote and protect the fundamental rights of those who cross the EU’s external borders. No incidents or complaints were reported by travellers using the app throughout the project. No complaints were received via voluntary survey forms or anonymous review options available in app stores.

### 6.3 Cybersecurity

The use of mobile devices for biometric enrolment introduces a broad threat landscape that includes unauthorised access attempts, interception of sensitive information during transmission, credential theft, presentation attacks against facial recognition systems, malware infections, device compromise through rooting or jailbreaking, backend service abuse, and denial-of-service attacks. To address these challenges, the system is designed following a cybersecurity-by-design approach. Sensitive operations involving biometric verification and travel document validation are executed within trusted backend environments rather than on user devices. Strong cryptographic protections safeguard data both at rest and in transit, while authentication and authorisation controls protect access to services. Additional measures include anti-tampering controls, device integrity verification, API throttling, secure communications, liveness detection mechanisms to mitigate presentation attacks, and continuous monitoring capabilities. The adoption of the OWASP Mobile Application Security Verification Standard (MASVS) provides a structured framework to ensure resilience against known mobile application threats. These measures not only protect the system against cybersecurity risks but also contribute to maintaining the integrity, reliability, and legal trustworthiness of the overall border management process.

From a cybersecurity perspective, the app was designed and tested using the Open Web Application Security Project (OWASP) Mobile Application Security Verification Standard (MASVS). ^28^ Testing was performed in the following areas.

• **MASVS-STORAGE **: Secure storage of sensitive data on a device (data at rest).
• **MASVS-CRYPTO **: Cryptographic functionality used to protect sensitive data.
• **MASVS-AUTH **: Authentication and authorisation mechanisms used by the mobile app.
• **MASVS-NETWORK **: Secure network communication between the mobile app and remote endpoints (data in transit).
• **MASVS-PLATFORM **: Secure interaction with the underlying mobile platform and other installed apps.
• **MASVS-CODE **: Security best practices for data processing and keeping the app up-to-date.
• **MASVS-RESILIENCE **: Resilience to reverse engineering and tampering attempts.

## 7. User acceptance and operational testing results

This section is divided into two distinct subsections, each serving a specific purpose. The first subsection outlines the user acceptance tests, and the second describes the operational tests, collectively facilitating a structured and multi-faceted evaluation of the project’s outcomes.

### 7.1 User acceptance test

One of the activities that took place before the piloting of the app in a real operational environment was a user acceptance test in April 2024 at Schiphol Airport. The goal of this test was to evaluate the pre-pilot release of the app to validate its readiness, including the design of the user interface, usability aspects, and technical implementation. Any findings from the user acceptance test could support the improvement of the app before the operational test at Arlanda Stockholm Airport in May and June 2024.

The version of the app during the user acceptance test was not integrated with any national backend or any systems based at Schiphol airport, and no data were submitted to any country backend. With support from the Frontex project team, travellers during an airport layover were offered the opportunity to voluntarily install the app on their own device and simulate the use of the app for their journey. While the traveller was using the app, the Frontex team was able to observe the user experience and assess the app usability, testing key features and functionalities, such as passport reading and face image verification. The initial set target was to engage at least 250 volunteer non-EU travellers (i.e., travellers that are subject to a visa requirement or visa-exempt travellers who do not hold a residence permit or similar) in the transit area of Schiphol airport. These travellers were selected because they are subject to the Entry/Exit System (EES) in the future.

More specifically, the purpose of the user acceptance test was to confirm the Quick Border app usability from an end-user perspective (operational ease-of-use), to confirm that the Quick Border app performs the intended functions (pre-register traveller identity – including liveness detection – and journey and submits data to a simulated national backend environment), to present consolidated communication material and check if messaging is clearly understood by the travellers, to collect feedback from participants via surveys and behaviour observation, and to collect and assess preliminary key performance indicators (KPIs) and check if they are fit for purpose.

The user acceptance tests involved more than 200 third-country national passengers travelling to and/or from different destinations in the extra Schengen Area of the Amsterdam Schiphol Airport. The tests were supported by specially delegated Royal Netherlands Marechaussee (RMN) airport employees and the Frontex project team. Test facilitators approached passengers waiting in the airport lounges, invited them to download the app, and supported them with the pre-registration process. Passengers who downloaded the application on their own devices were asked to provide feedback on their user experience, app usability, key features, and functionalities such as passport reading and face image verification.

The figures related to the UAT are:

• 350+ app downloads.
• 204 Travellers scanned passports (VIZ capture),
• 178 located the chip in their passport (NFC capture),
• 85 took the selfie,
• 85 Liveness checks were performed,
• Twenty nationalities, the top three nationalities that took part in the voluntary tests: (1) the United Kingdom, Great Britain, and Northern Ireland (25%), and (2) New Zealand (15%) and the USA (9%).

As the app was not integrated into any national system, the process finished before submission with a message of appreciation for the volunteer’s effort.

The travellers were asked to provide feedback on the application using a start rating system (5 stars maximum) and provided the following average rating assessments for different aspects of the app:

• App as a whole: 4.53
• Ease of navigation: 4.47
• Passport scanning/reading: 4.11
• Facial image capture: 4.25
• Entry/Exit questionnaire: 4.42
• Likelihood of recommending the app: 4.42
• App as a tool to facilitate travelling: 4.45

Additionally, travellers shared their ideas on application improvements, desired more information about the airport facilities available in the application, and identified features that should be available in the application.

Following the UAT, a new Release 4.2 was issued, including more than 30 fixes and improvements to the workflow and user experience, based on recommendations from the Schiphol airport tests. Moreover, the new app version includes enhanced error messages, improved stability, extensive FAQs, better action button placement, cleared questions, and more precise instructions. This allowed the delivery of the required changes for the operational test.

### 7.2 Operational test

The operational test of the app took place from May 21th to June 28 ^th^ with real travellers at Sweden’s Stockholm Arlanda airport.

To reach these travellers, a collaboration was established with airlines operating flights arriving in Arlanda from outside the Schengen Area, including the Scandinavian Airlines (SAS) and Emirates. The airlines invited travellers to voluntarily participate in the operational test using an email campaign, with SAS sending 3,711 and Emirates sending 7,286 email invitations. The click-to-open rate, which was monitored only using SAS, was 25%. In the invitation, e-mail travellers were provided with an access code, a functionality implemented to restrict the app to the invited travellers.

During the operation pilot, the app was downloaded 4,840 times, with 75% of the downloads coming from iOS devices and 25% from Android. The achieved average rating was very high, 4.25 and 4.4 respectively in iOS App Store and Google Play. Despite some technical challenges for app installation using the access code and understandability of travellers about the app goals and functionality, the campaign revealed the potential for significant user engagement and provided valuable insights for future improvements.

During the operational test, over 3,188 travellers started the pre-registration process using the app, and 1600 completed all registration steps, including submission of the data to the national backend and crossing of the border using the pre-registered data. Travellers were from 50 different countries, with the majority being from the United Kingdom, the United States, Canada, and Australia (75%).

**Figure 4.**
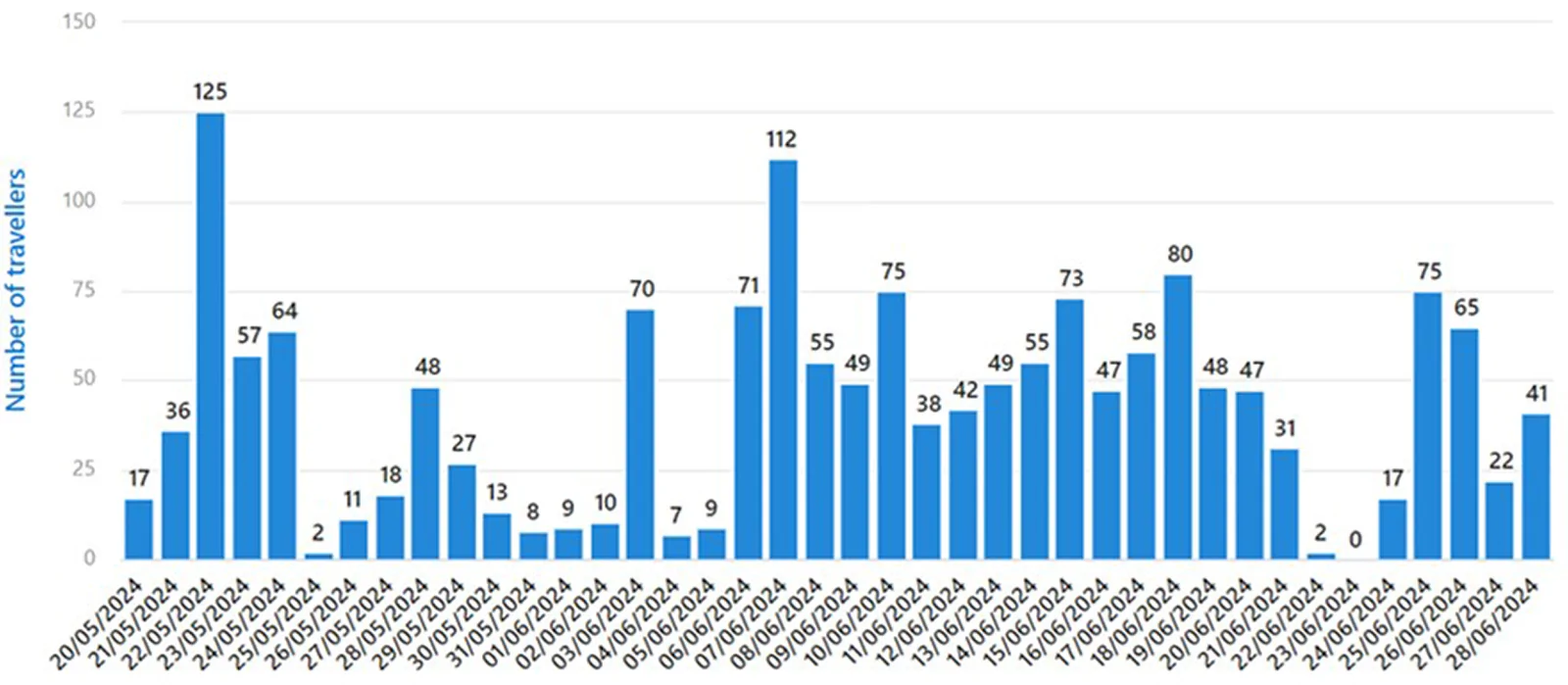
Number of travellers enrolled on a daily basis.

The daily enrolment rate of travellers utilizing the application during the operational period at Arlanda Airport is shown in Figure 4. These numbers include travellers registering at home before starting the journey and travellers who were invited to register at the airport after landing in Arlanda.

**Figure 5.**
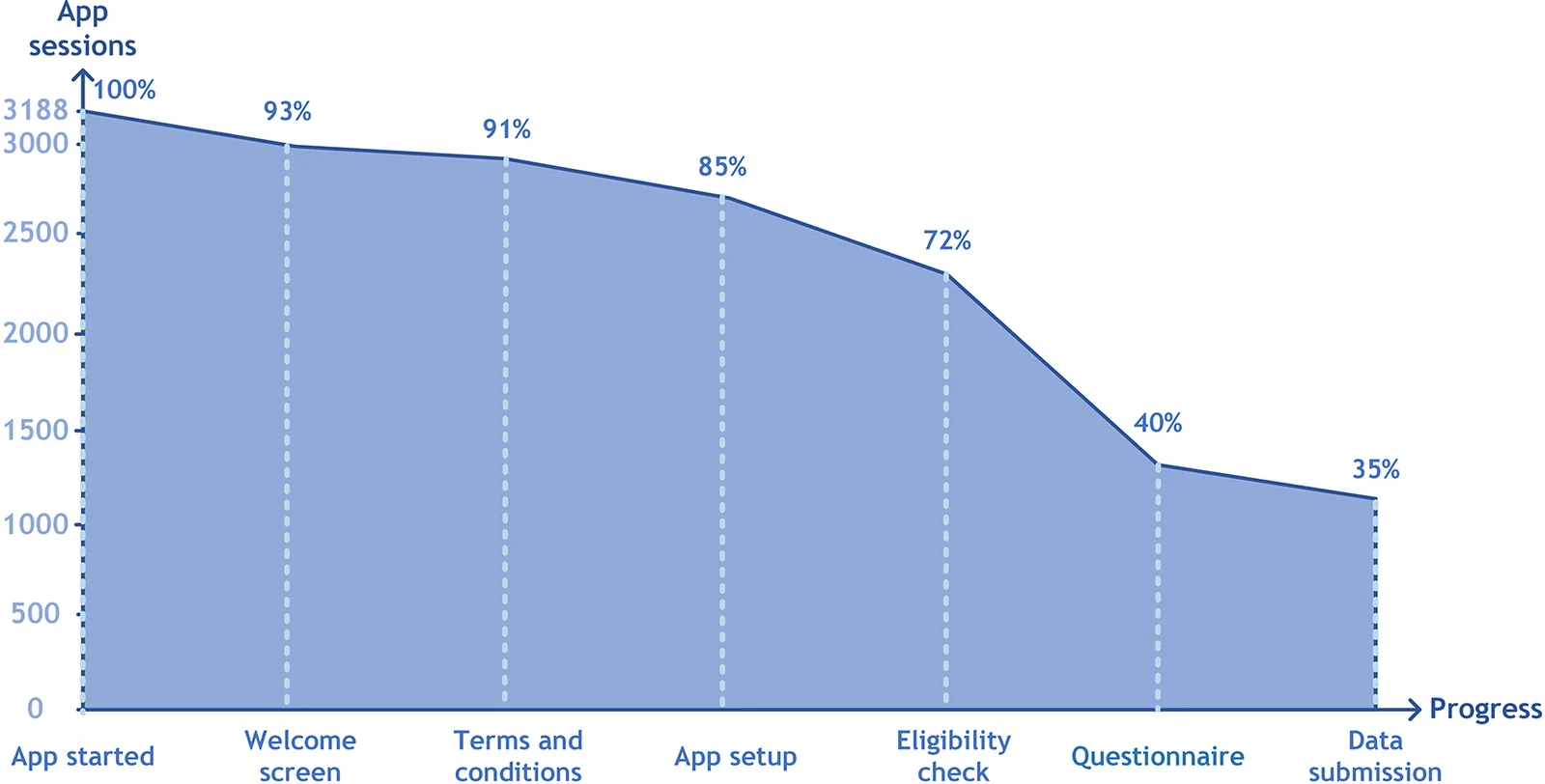
User retention/drop after each step of the process implemented in the app.

As depicted in Figure 5, 3,188 mobile app sessions were created, meaning that a user had installed and started the app with the creation of a session identifier in the app backend. From this total number, 93% reached the welcome screen and proceeded to the terms and conditions that 91% accepted. The app setup process was completed by 85% of the participants, and 72% passed the eligibility check. However, there was a noticeable drop between the eligibility check and questionnaire stages. This is a point in the process during which passenger document reading, facial image acquisition, and facial verification take place. As a result, by the time users reach the questionnaire stage, only 40% are left to complete. Ultimately, 35% of participants completed the entire process. The overall number of travellers was larger than the number of sessions, as many sessions included more than one traveller.

**Figure 6.**
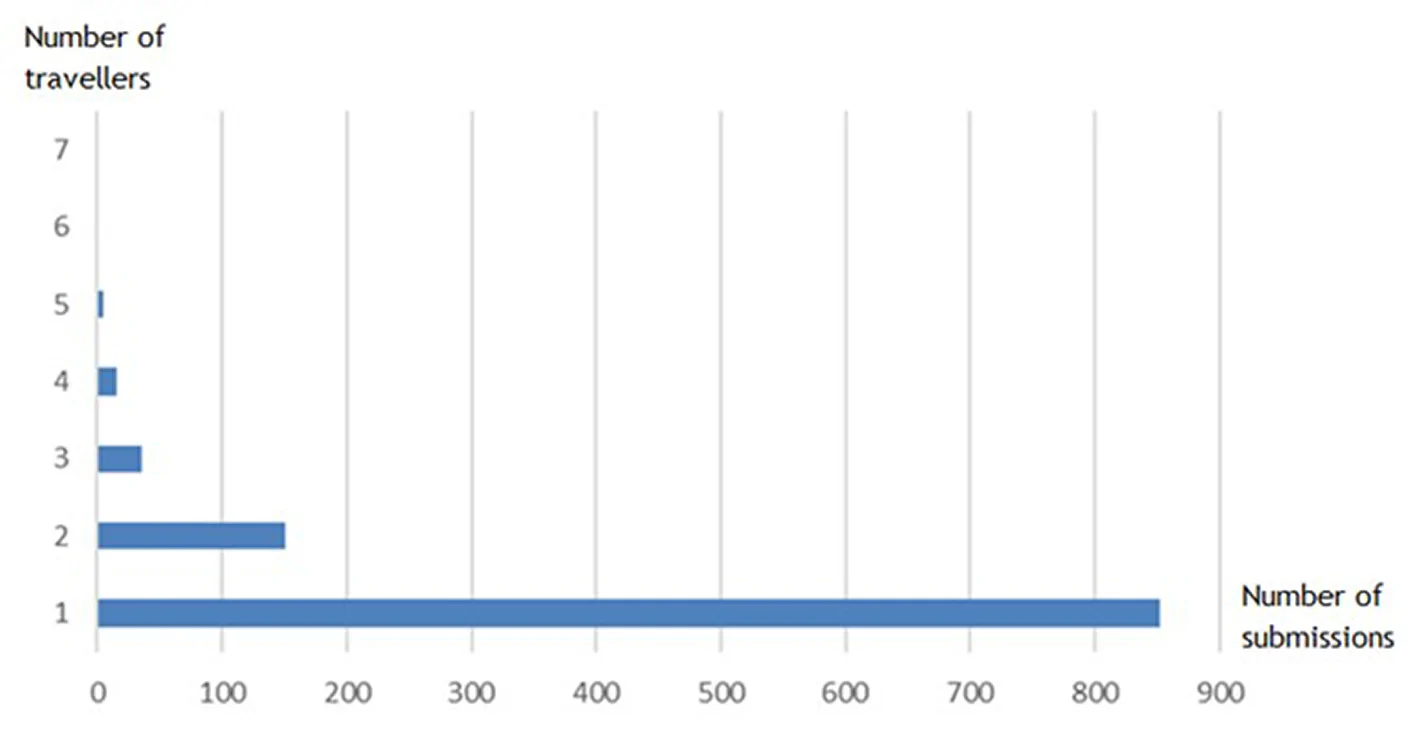
Number of travellers in each journey submission using the app.

The eligibility and questionnaire steps are measures for each app session, which represent multiple travellers going through these steps in a specific session. Figure 6 shows the number of travellers added for each data submission performed by the app. Approximately 850 submissions included only one traveller, while 150 and 30 submissions included two and three travellers, respectively. Very few submissions also included three to seven travellers, suggesting that the app has been used to submit information from large groups of travellers, including families, and could also be used to facilitate the border crossing of large groups in organised trips.

**Figure 7.**
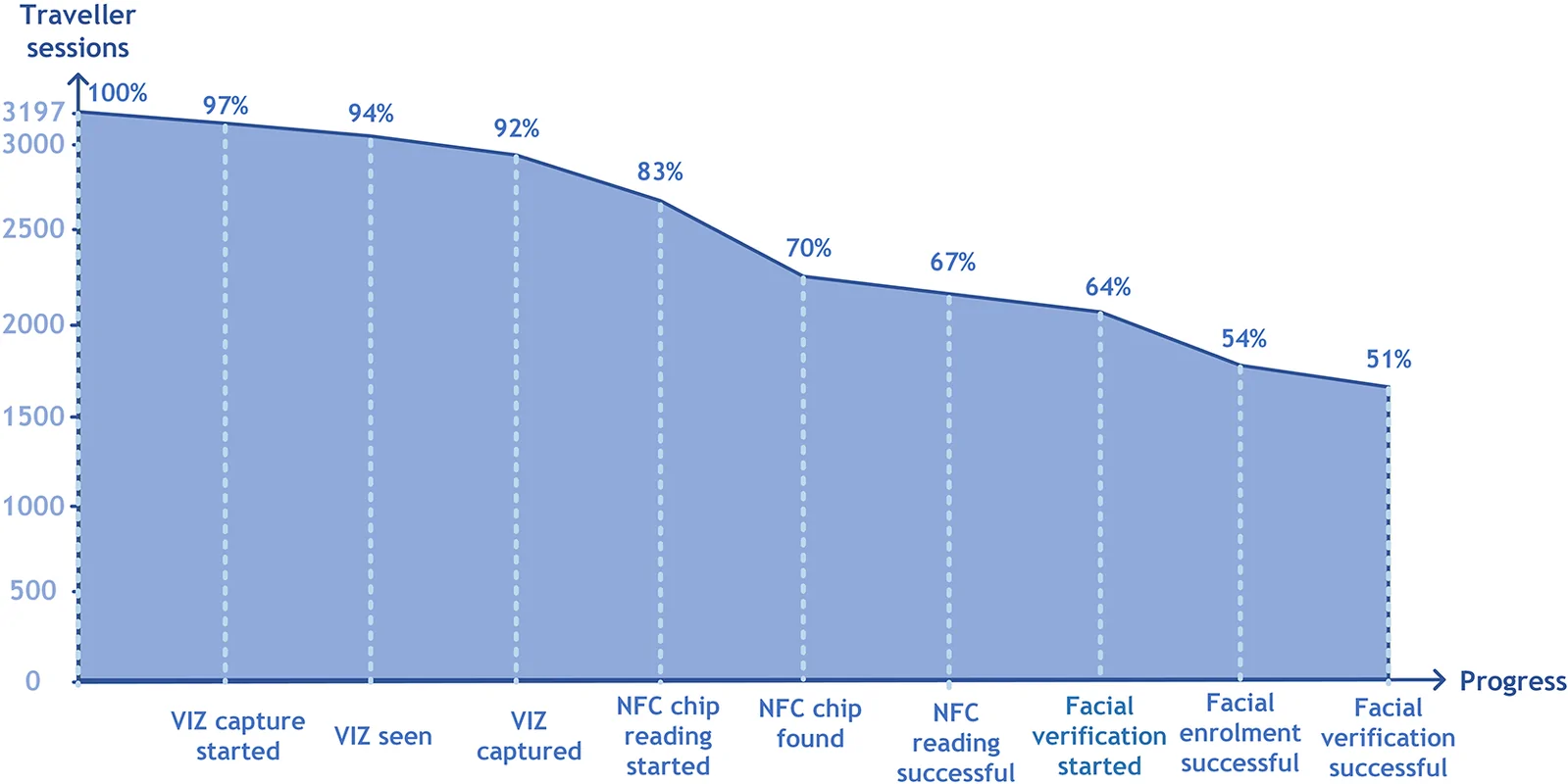
User retention/drop after each step of the eMRTD reading and biometric enrolment/verification.

Figure 7 shows the user retention and drop rate after each step of the eMRTD reading and biometric enrolment/verification. The overall user retention from all travellers that started the VIZ capture is around 50%.

**Figure 8.**
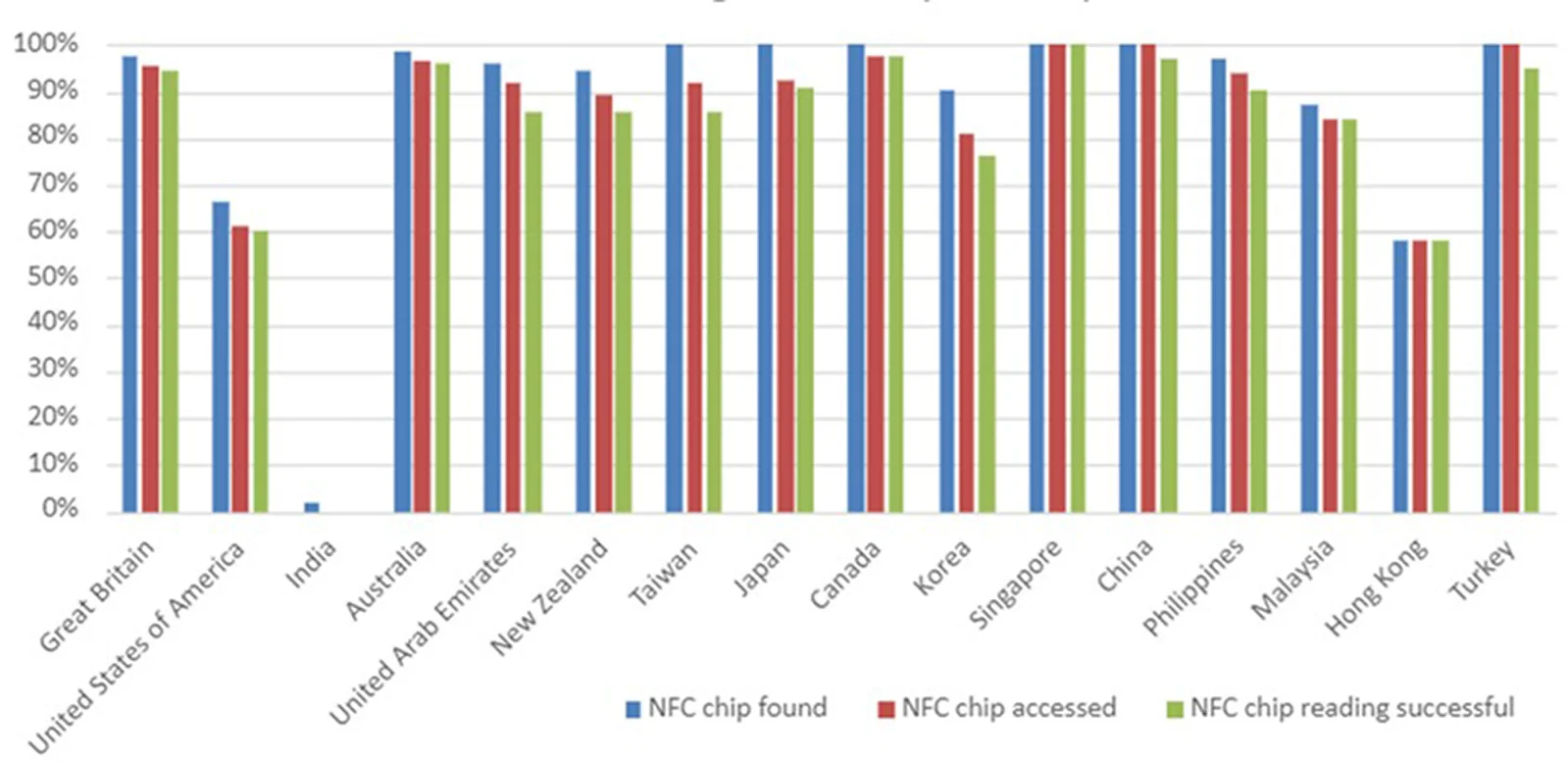
NFC reading success by country.

Figure 8 shows the NFC reading success rate by country. Overall, in most countries, the success rate is very high (above 90%). For the United States, the success rate was significantly lower considering the location and protection features of the passport chip, as the cover of the passport is shielded, and the chip can only be read with the passport page open. This feature introduces a few issues for travellers. Furthermore, travellers from India could not use the app because their passport did not contain a chip. Finally, travellers from Hon Kong experience issues with the reading of the eMRTD chip in a few cases, which could indicate interoperability issues with the library used by the mobile app to access and read the chip information.

**Figure 9.**
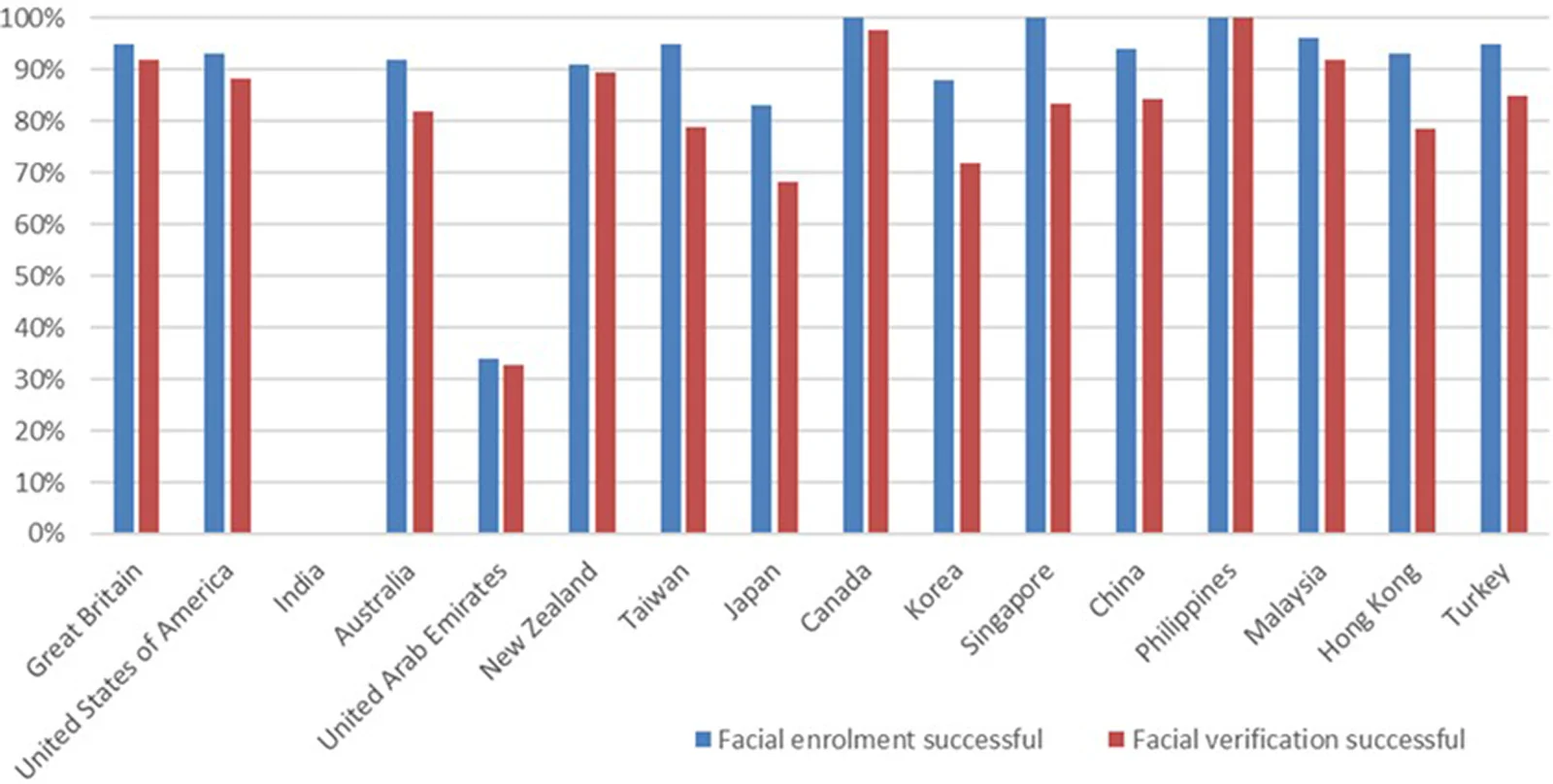
Facial verification success by country.

Figure 9 presents the success rates for countries with facial enrolment and verification. In this case, most countries also had a very high success rate of over 85%. For the United Arab Emirates, the success rate was lower for Japanese and Korean travellers. Because of the low number of travellers in these categories using the app, a general conclusion for the lower success rate cannot be drawn.

**Figure 10.**
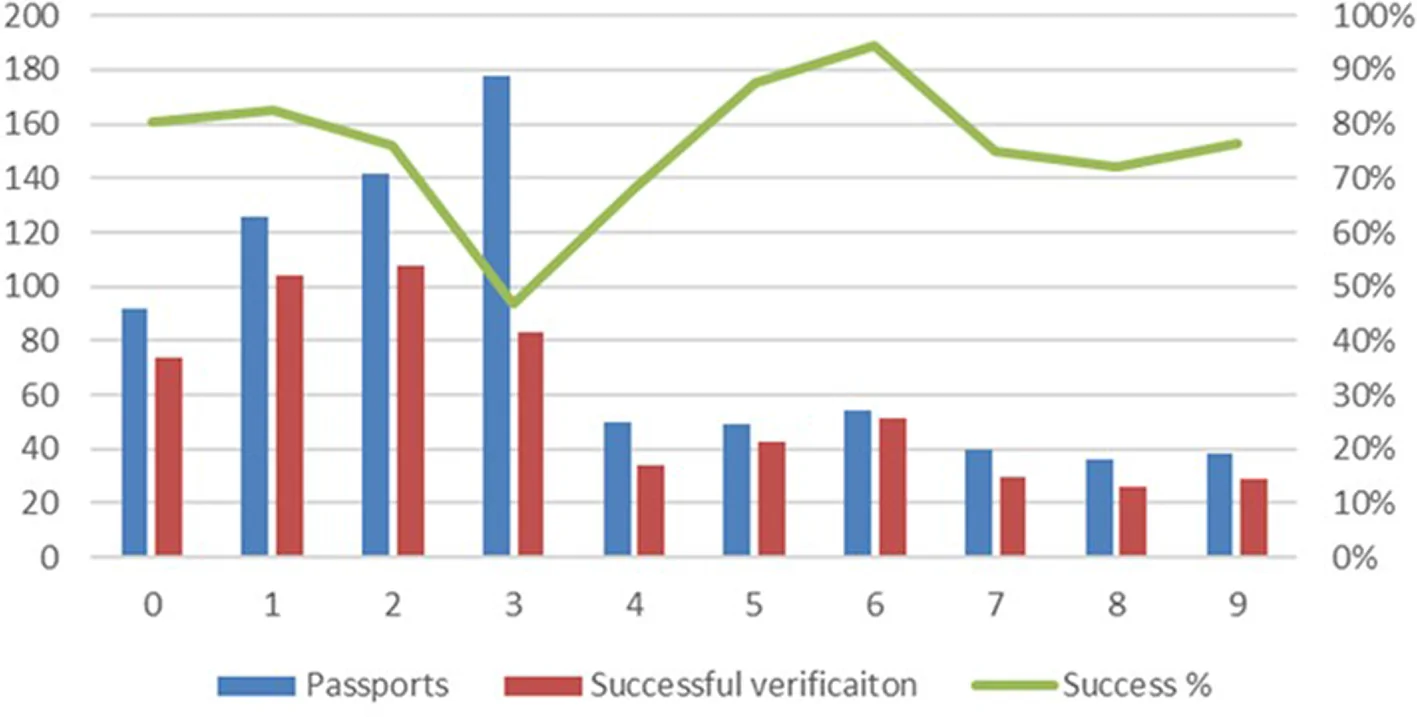
Passport age and face verification success rate.

**Figure 11.**
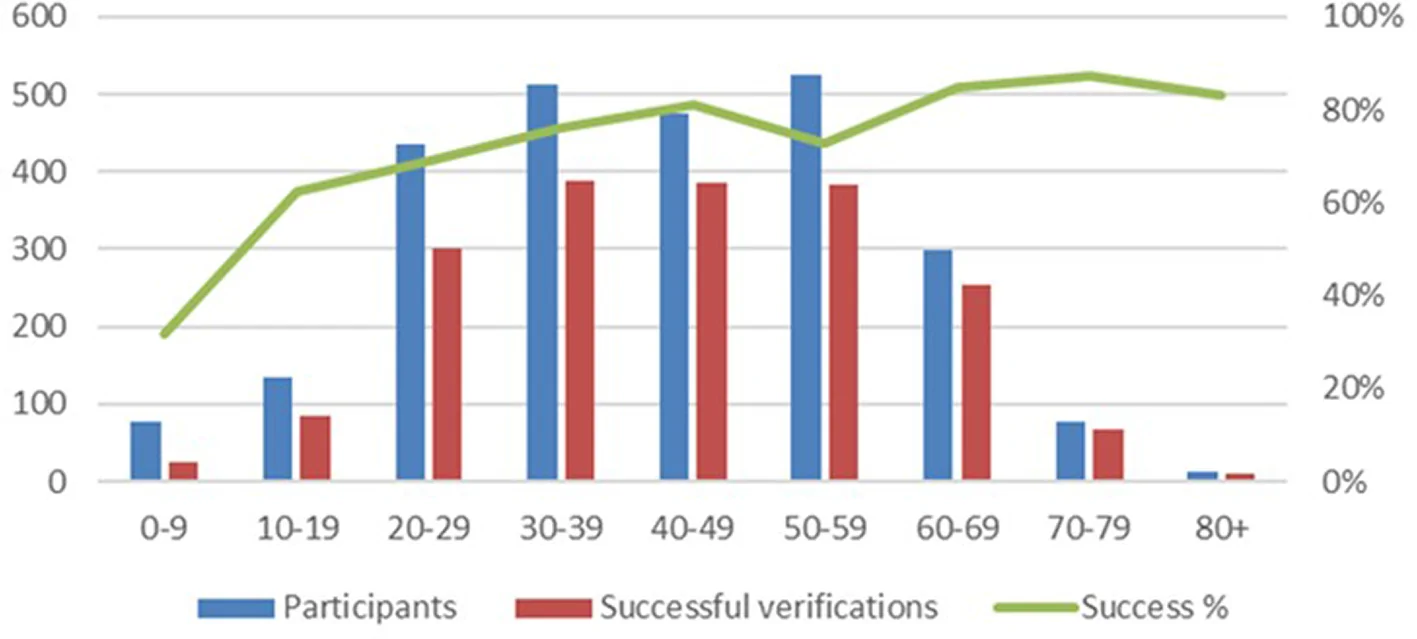
Age distribution of traveller and face verification success rate.

Figures 10 and 11 present the verification success rate considering the age of the travel document and traveller. A lower success rate was observed for 3 years old passports, which could be caused by passports issued during the COVID 19 pandemic with lower-quality pictures. The verification success rate was also slower for younger travellers (below 10 years of age), which is expected since children change significantly after a few years and it is more challenging to enroll good-quality pictures in their travel documents.

**Figure 12.**
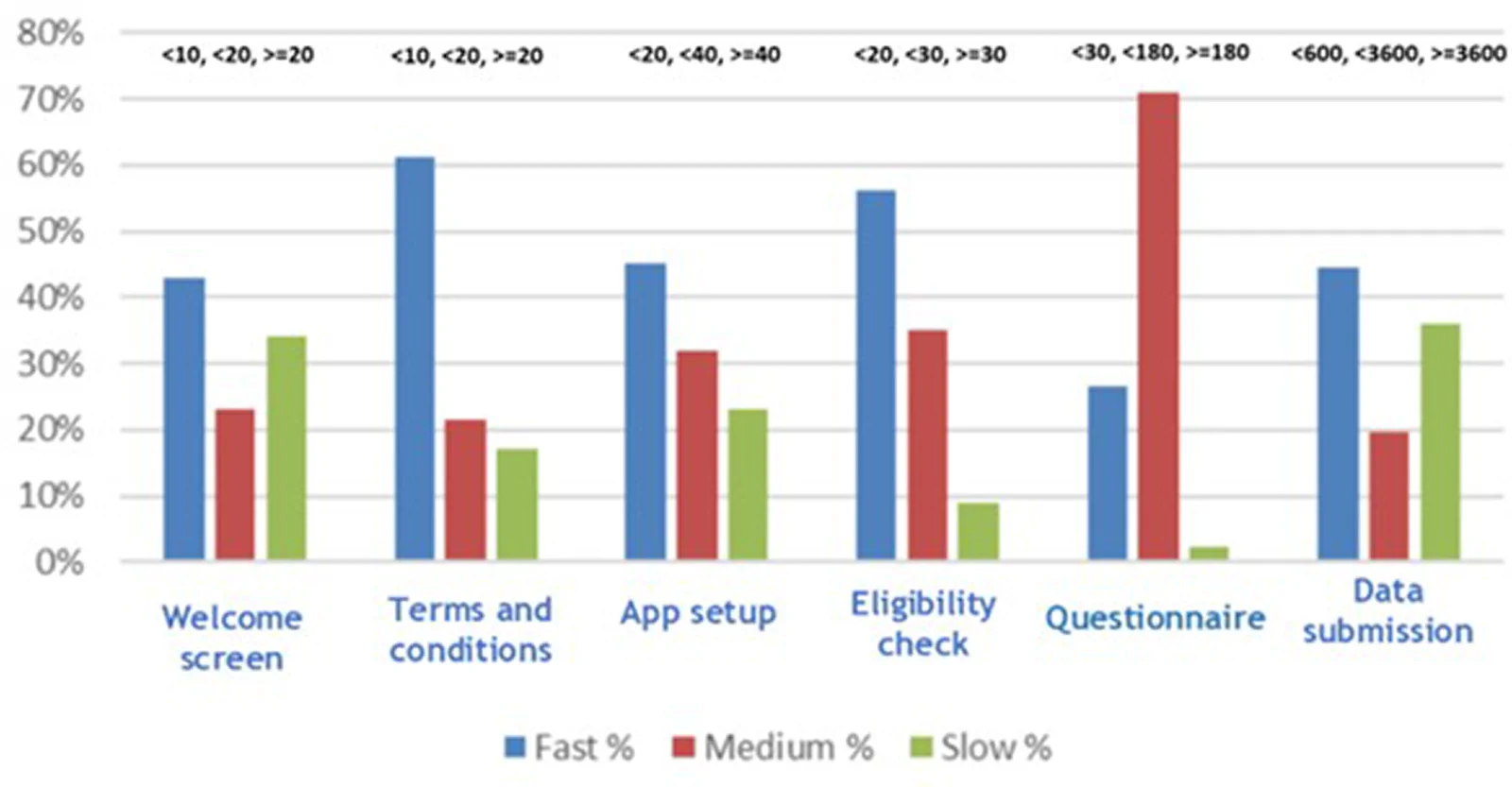
Distribution of processing speeds in the application processes.

Finally, the following figures present an outline of the performance KPIs captured during the piloting at Arlanda, focusing on both the speed of executing various processes during app usage, as well as the impact on the border crossing times for visa-required and visa-exempt travellers. As shown in Figure 12, over 60% of users quickly completed the welcome screen, Terms and Conditions, with the Eligibility and Setup stages also seeing fast completions. Conversely, the Questionnaire stage required more time, with over 70% of users taking a medium amount of time, and the data submission takes significantly longer because users can save and decide to submit the journey data at a later stage. For each stage, the mapping between fast, medium, and low in absolute terms (seconds) is also described.

**Figure 13.**
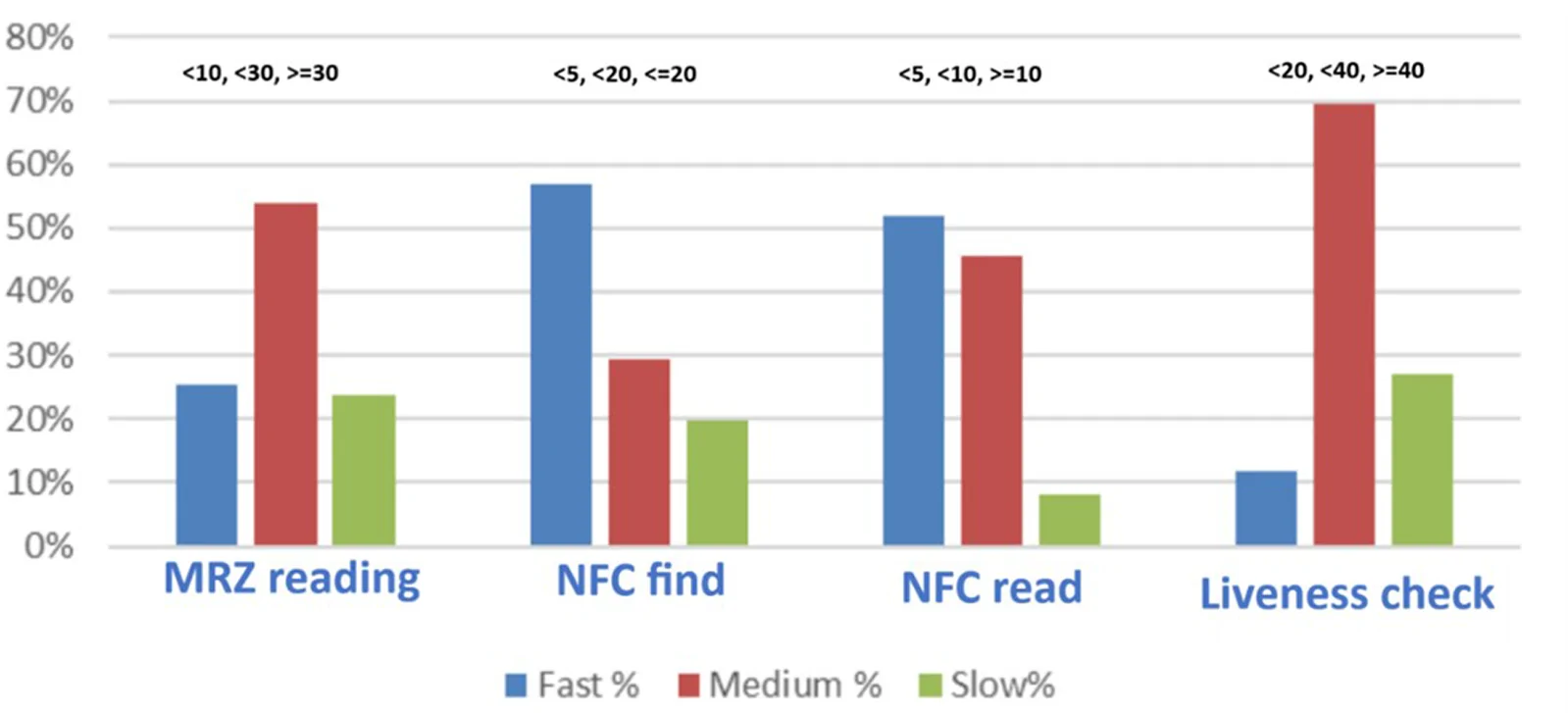
Distribution of processing speeds in document processes & liveness check.

In Figure 13, the system processing speeds highlight user interactions during the document reading and facial verification stages. Regarding document registration, over 50% of users quickly completed the NFC find and read processes, whereas the MRZ reading stage saw most users taking a medium amount of time. The Liveness Check stage was slower, with around 70% of users in the medium-speed category, likely due to the inherent time requirements of the process.

**Figure 14.**
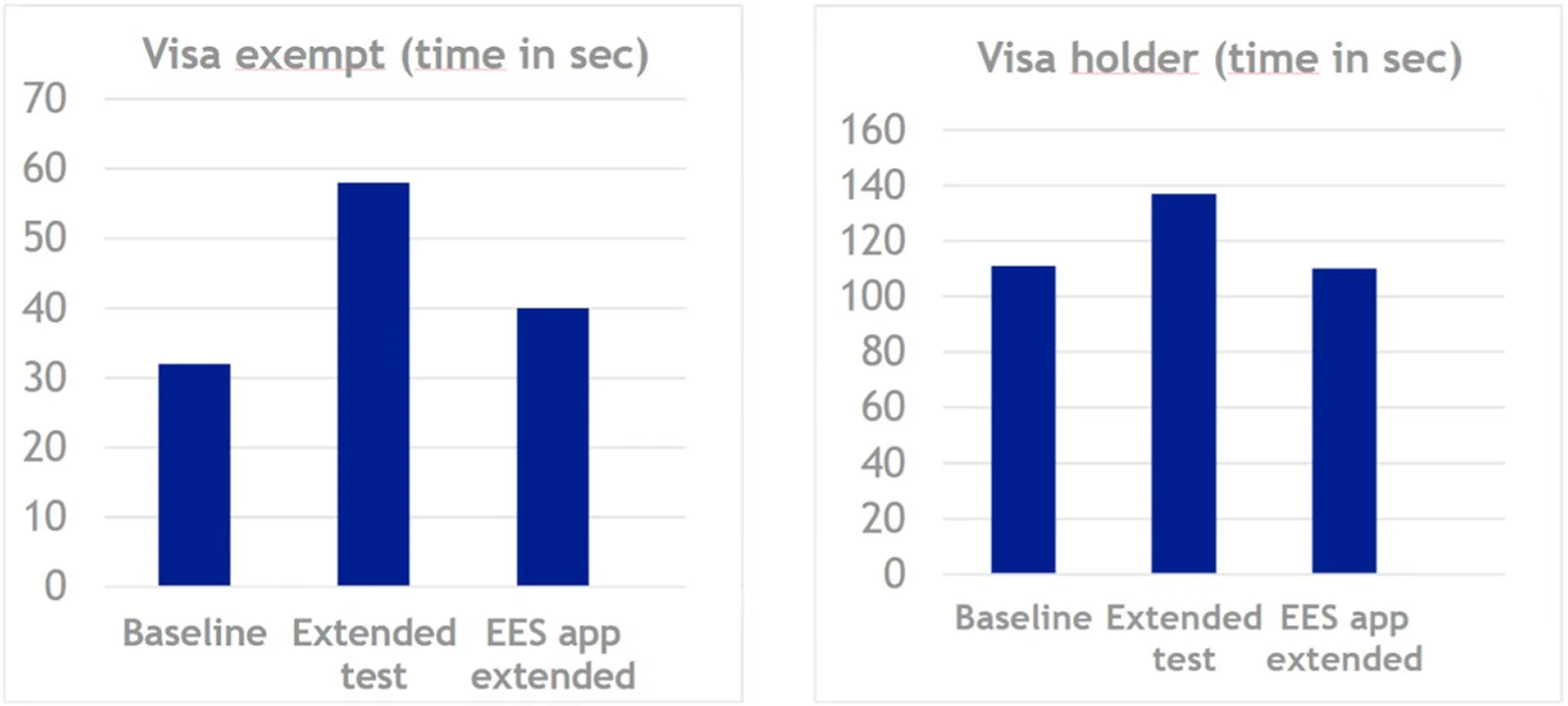
Comparison of traveller processing time at Arlanda for Visa exempt and holders.

7.2.1 Impact on border crossing times

During the period leading up to the pilot project and during the Arlanda operations phase, the Swedish authorities carried out extended tests to assess the system’s preparation for entry into operation of the EES and traveller processing times at the border. The data from these tests were provided to the project as consolidated input for a baseline, considering the regular border check process, extended EES test, and EES app extended test.

The baseline time considers the standard border check as performed in a manual booth without considering any step introduced by the EES, therefore excluding the biometric data capture for visa-exempt travellers and only fingerprint verification for visa-required travellers. The extended EES test considers the simulation of relevant parts of the border crossing process, as will be conducted after the entry into operation of EES with biometric capture and enrolment at the border only for the facial image. The EES app-extended test is the processing time when the App4EES is used, considering the pre-enrolment of biometrics and provision of additional information in the application (questionnaire), with data reconciliation at the manual booth. It should be noted that these tests were performed both to help prepare for EES entry into operations and to provide a baseline value that could be used to assess App4EES potential impact.

As depicted in Figure 14, the border processing time improvement using App4EES was on average 25% for both Visa exempt and Visa holders. App4EES mitigates the impact on the border processing time, bringing the processing time down to the baseline time, thereby significantly reducing the overhead of the EES processes.

### 7.3 General discussion, observations, and lessons learnt

The operational assessment of the App4EES pilot project provided a unique opportunity to evaluate the feasibility of mobile pre-registration under realistic border control conditions. The combination of quantitative performance data, user feedback, and qualitative observations enables a comprehensive discussion of the operational, technical, and human factors of the system.

From an operational standpoint, the results demonstrate that traveller pre-registration through a mobile app can significantly reduce processing times at border-crossing points without compromising the integrity or security of the process. The measured 25% improvement in average processing time for visa-exempt travellers represents a meaningful efficiency gain when extrapolated to large-scale border operations. These findings confirm that offloading parts of the enrolment and verification workflow from the border counter to the traveller’s device can reduce the cognitive and procedural burden on border guards while maintaining compliance with the Entry/Exit System (EES) requirements.

Several observations emerged regarding traveller behaviour and interaction with the app. First, user acceptance was high, as evidenced by positive satisfaction ratings and consistent completion rates across the demographic groups. Nevertheless, some users experienced difficulties in specific steps, particularly in the near-field communication (NFC) passport chip reading and liveness detection phases. These difficulties were often attributed to limited user familiarity with NFC use and environmental lighting conditions, rather than technical faults. Future iterations of the app could benefit from enhanced on-screen guidance, visual feedback, and context-sensitive error handling to reduce the drop-off rates in these stages.

The inclusion of multi-traveller functionality proved to be a decisive factor in user engagement, particularly for families and organised groups. This capability enables the aggregation of multiple individual registrations into a single transaction, reducing the time and effort required for collective travel parties. The data suggest that multi-traveller submissions not only increase convenience, but also demonstrate the scalability of the mobile enrolment model for diverse traveller profiles.

From a technical and cybersecurity perspective, the architecture adopted—featuring a trusted backend environment and strict separation between user devices and national systems—proved to be robust and resilient. Adherence to the OWASP MASVS framework ensured that no significant vulnerabilities were detected during or after the pilot study, confirming the suitability of the security-by-design approach. Similarly, the Comprehensive Data Protection Impact Assessment (DPIA) validated compliance with the General Data Protection Regulation (GDPR) and demonstrated that privacy-preserving mechanisms can coexist with operational efficiency.

The pilot study also revealed valuable lessons regarding stakeholder coordination and process integration. Effective collaboration between Frontex, the participating Member States, airport authorities, and airline partners was essential to achieving interoperability and ensuring smooth data flow between systems. This experience underscores the need for early alignment of operational procedures and communication strategies among stakeholders, particularly when introducing new digital pre-registration tools within the complex regulatory environment of EU border management.

Finally, it was observed that travellers’ education and communication are pivotal to achieving optimal performance. The clarity of the app’s purpose, data usage explanations, and step-by-step instructions directly influence user confidence and engagement. Future deployment strategies should, therefore, include comprehensive public information campaigns and integration with airline and travel-booking ecosystems to maximize adoption.

## 8. Conclusions and Lesson learning/directions

The App4EES pilot project has successfully demonstrated the feasibility and effectiveness of a mobile app solution for pre-registering biometric and biographic data to enhance border-crossing efficiency in a real operational environment. The project outcomes show that the app can be used by a diverse group of travellers, with excellent results in terms of user experience, efficiency, and data protection. This conclusion is supported by the high number of participants and the positive feedback received during the testing phase, which suggests that the app has the potential to be widely adopted and used at various border-crossing points.

The app was successfully tested in a real operational environment with a high diversity of real users, demonstrating its effectiveness and usability in a practical setting. The project’s results show that the app can be used by travellers from different countries with different types of travel documents and varying levels of familiarity with mobile apps.

The global results showed significant time savings for most travellers, with an average reduction of 30 s in border-crossing times for visa-exempt travellers, which can lead to improved efficiency and reduced waiting times at border-crossing points.

A key element from the beginning of the project was the design with data protection and cybersecurity in mind, ensuring that travellers’ personal data are secure and protected. The project’s outcomes show that the app meets the requirements of the General Data Protection Regulation (GDPR) and other relevant data protection regulations and that it has been aimed at minimizing data breaches and other security threats.

The app’s user interface and user experience have been positively evaluated by participants, with an average overall rating of 4.25/5.00 on Android and 4.40/5.00 on iOS stores, which suggests that the app is user-friendly and easy to use.

Future research should focus on improving the interoperability between pre-registration systems and national border infrastructures through standardised APIs and harmonised data formats. Incorporating emerging technologies such as digital travel credentials (DTCs), verifiable credentials, and mobile contactless fingerprint capture could further streamline the pre-registration process and reduce the dependency on hardware at border checkpoints. Moreover, expanding support for offline operations and multilanguage accessibility could enhance inclusiveness and usability for travellers from diverse backgrounds. Scaling the system from the pilot level to a pan-European deployment requires a unified governance model and the establishment of common operational guidelines for Member States. The experience gained suggests that the early involvement of national authorities and border personnel is critical to aligning the pre-registration workflow with existing border management procedures. Developing an EU-level framework for the certification and quality assurance of mobile pre-registration applications could foster trust and ensure technical consistency across borders.

The project outcomes have contributed to the development of a robust and user-friendly mobile app solution for border management, which can be further enhanced and expanded to include additional features and functionalities, such as the use of digital travel credentials and advanced biometric technologies such as mobile contactless fingerprints. The App4EES pilot project has demonstrated the potential of mobile app solutions to enhance border-crossing efficiency, improve user experience, and ensure data protection and cybersecurity, and has contributed to the development of a robust and user-friendly mobile app solution for border management.

## Ethics and consent

Ethical approval and consent were not required.

## Data Availability

The datasets generated and analysed during this study cannot be shared due to legal and ethical restrictions. The operational pilot involved the collection of personal and biometric data from volunteer travellers, including facial images and travel document information. These data constitute sensitive personal data under the General Data Protection Regulation (GDPR) and were collected exclusively for the purpose of conducting the App4EES operational pilot. Participation in the pilot was based on explicit informed consent, under the condition that the data would be used only for the evaluation of the pilot project and retained for a limited period. In accordance with the data minimisation and storage limitation principles of the GDPR, and as defined in the project’s Data Protection Impact Assessment (DPIA) reviewed by the Frontex Data Protection Office, the datasets containing personal information were securely deleted after completion of the evaluation phase. Therefore, the original data contain identifiable biometric and travel document information are no longer retained by the project and, consequently, cannot be made available.

## Footnotes

[1]
Frontex EES Land Border Pilot Project: Bulgaria and Spain Final Evaluation Report chapter 8.
