# Supplementary material for: Operational assessment of a border management mobile app for traveller pre-registration
Source: Open Res Eur. 2026 Jul 28;6:160. Originally published 2026 May 11. [Version 2] doi: 10.12688/openreseurope.22737.2 (PMC13560578; doi:10.12688/openreseurope.22737.2)
Supplement: Peer review history

# Reviewer report: Reviewer response for version 2

**DOI:** 10.21956/openreseurope.26448.r78124

**Published:** 2026-08-25

**Authors:** Javier Galbally

**Affiliations:** 1 EU Agency eu-LISA, Tallinn, Estonia

After going through the changes/revisions made by the authors, in my view, the article is ready to be indexed

Is the study design appropriate and does the work have academic merit?

Partly

Is the work clearly and accurately presented and does it cite the current literature?

Yes

If applicable, is the statistical analysis and its interpretation appropriate?

Yes

Are all the source data underlying the results available to ensure full reproducibility?

No

Are the conclusions drawn adequately supported by the results?

Yes

Are sufficient details of methods and analysis provided to allow replication by others?

Yes

Reviewer Expertise:

NA

I confirm that I have read this submission and believe that I have an appropriate level of expertise to confirm that it is of an acceptable scientific standard.

# Reviewer report: Reviewer response for version 2

**DOI:** 10.21956/openreseurope.26448.r78125

**Published:** 2026-07-31

**Authors:** Nikolaos Pitropakis

**Affiliations:** 1 Department of Computing and Mathematics, The American College of Greece, Aghia Paraskevi, Greece

All comments have been addressed.

Is the study design appropriate and does the work have academic merit?

Yes

Is the work clearly and accurately presented and does it cite the current literature?

Yes

If applicable, is the statistical analysis and its interpretation appropriate?

Yes

Are all the source data underlying the results available to ensure full reproducibility?

No source data required

Are the conclusions drawn adequately supported by the results?

Yes

Are sufficient details of methods and analysis provided to allow replication by others?

Partly

Reviewer Expertise:

cybersecurity, cloud computing security, adversarial machine learning

I confirm that I have read this submission and believe that I have an appropriate level of expertise to confirm that it is of an acceptable scientific standard.

# Reviewer report: Reviewer response for version 1

**DOI:** 10.21956/openreseurope.24593.r74212

**Published:** 2026-05-28

**Authors:** Javier Galbally

**Affiliations:** 1 EU Agency eu-LISA, Tallinn, Estonia

The paper is well written and structured with no major flaws.

 The paper does not have any significant contribution from a scientific point of view. Therefore, if the main purpose of the journal is to present new developments in science, the paper, even though has no mistakes and the quality is high, may not be suited for indexing . 

 However, it has to be highlighted, that the paper does present some valuable insights from a pure operational perspective that can contribute to improve the impact of EES on travellers and to help the general deployment of the system. Therefore, if the purpose of the journal is not only purely scientific, but also to present results from practical field/proof of concept projects, the present paper merits in my view without question being published.

 A minor point of improvement. The state of the art is well presented, with other similar apps to the App4EES described in the paper properly described. However, the distinguishing factors of App4EES with respect to those other apps are not clearly explained. Why is there the need for a different app? What is “unique” about it with respect to the other apps both from a technical and operational perspective ?

 In the intro EES is presented with a rather limited scope: "The Entry/Exit System (EES)3 is a new system implemented by the European Union to enhance security and information sharing across European Member States." 

 The main purpose of the EES is to substitute the current control of entries and exits of the Schengen area through the manual stamping of passports, to an automated digital system: https://travel-europe.europa.eu/ees/what-is-the-ees

 https://www.eulisa.europa.eu/activities/large-scale-it-systems/ees

 It would be important to highlight that the EES is designed/developed with a two-fold objective:

 - Indeed, as indicated in the paper, enhance security and through:

 Preventing Irregular Migration: The EES will help track who comes in and out of the Schengen Area, using fingerprint and face data to stop people from overstaying, using fake identities or misusing visa-free travel.

 Increasing Security in the Schengen Area: The EES will give border officers and law enforcement authorities access to important traveller information, helping them to spot security risks and support the fight against serious crimes and terrorism.

 - BUT ALSO, help border crossing making the improving the traveller experience:

 Making Border Checks More Modern and Efficient: The EES will gradually replace passport stamps with a digital system that records when travellers enter and exit, making border checks faster and helping staff to work more efficiently.

 Making Travel Across Borders Easier and Faster: With EES, travellers will spend less time at the border thanks to faster checks, self-service options, and the possibility to give their information in advance.

 The way it is presented at the moment it seems that the EES is a system only for security purposes that will make the travellers experience more difficult and that will somehow make the Schengen area a less welcoming place.

 This is not to say that everything is solved and that there is room no for improvement. In fact, the mobile app presented in this paper is a step towards the second objective of making the EES work more seamlessly for travellers and helping the flow of visitors.

 MINOR COMMENT: British English vs American English spelling should be checked throughout the document. Examples:

 Traveller vs taveler

 Authorisation vs authorization

Is the study design appropriate and does the work have academic merit?

Partly

Is the work clearly and accurately presented and does it cite the current literature?

Yes

If applicable, is the statistical analysis and its interpretation appropriate?

Yes

Are all the source data underlying the results available to ensure full reproducibility?

No

Are the conclusions drawn adequately supported by the results?

Yes

Are sufficient details of methods and analysis provided to allow replication by others?

Yes

Reviewer Expertise:

Biometric recognition, data science, border management, identity management

I confirm that I have read this submission and believe that I have an appropriate level of expertise to confirm that it is of an acceptable scientific standard, however I have significant reservations, as outlined above.

# Response

**Published:** 2026-06-24

**Authors:** Joanna Darmos

**Affiliations:** Research and Innovation, European Border and Coastguard- Frontex, Warsaw, Poland

*Dear Reviewer,* *We sincerely thank you for your positive assessment of the manuscript and for recognizing both the quality of the work and the operational value of the results presented. We appreciate your thoughtful comments and have revised the manuscript accordingly. Detailed responses are provided below.*

***Comment 1*** *"The paper does not have any significant contribution from a scientific point of view... However, it does present some valuable insights from a pure operational perspective..."*

***Response:*** *We thank the reviewer for this important observation. We agree that the primary*

*contribution of this work is not the introduction of a novel biometric algorithm, cryptographic method, or border management technology in isolation. Rather, the contribution lies in the design, deployment, and large-scale operational assessment of an integrated mobile pre-registration solution under realistic Entry/Exit System (EES) conditions.* *To better clarify the nature of the contribution, we have revised the Introduction and explicitly added the following sentences:* *The key scientific and operational contributions of this work are summarized below:*

- *Presentation of the first large-scale operational assessment of a mobile application for traveller pre-registration under Entry/Exit System (EES) conditions, involving more than 1,600 travellers in a real airport environment.*
- *Development and validation of an integrated pre-registration solution combining biometric enrolment, liveness detection, electronic passport verification, and conditions-of-entry questionnaires within a single mobile platform.*
- *Introduction and assessment of a multi-traveller registration capability supporting families, minors, and organised travel groups.*
- *Demonstration of measurable operational benefits, including an average reduction of approximately 30 seconds in border processing times and a 25% improvement in efficiency compared with standard EES testing procedures.*
- *Design and validation of a privacy-preserving and cybersecurity-by-design architecture compliant with European legal and regulatory requirements.*
- *Provision of practical lessons learned and operational recommendations for future deployment of mobile pre-registration solutions across EU external borders.*

 
*We believe these modifications better position the paper as a contribution that combines applied research, operational validation, and evidence-based assessment of innovative border management technologies.*  

***Comment 2*** *"The distinguishing factors of App4EES with respect to those other apps are not clearly explained. Why is there the need for a different app? What is unique about it with respect to the other apps both from a technical and operational perspective?"*

***Response:*** *We appreciate this observation and agree that the differentiating characteristics of App4EES should be made more explicit. To address this point, we have added a comparative table in the State-of-the-Art section.*    

***Comment 3*** *"The way EES is presented at the moment seems that the EES is a system only for security purposes..."*

***Response:*** *We thank the reviewer for this valuable remark and fully agree that the original wording could unintentionally convey an incomplete view of the objectives of the Entry/Exit System (EES).* *The Introduction has therefore been revised to better reflect the dual purpose of EES. We believe this revision provides a more balanced presentation of EES and better contextualizes the role of the proposed solution within the broader modernization of European border management.* *Replace the current sentence:* *"The Entry/Exit System (EES) is a new system implemented by the European Union to enhance security and information sharing across European Member States."* *with:* *"The Entry/Exit System (EES) is a large-scale information system developed by the European Union to modernize the management of external borders through the digital registration of entry and exit events of third-country nationals. The system pursues a dual objective. First, it aims to strengthen security by helping to detect irregular migration, identify overstayers, prevent identity fraud, and support law-enforcement activities. Second, it seeks to improve the efficiency and quality of border management processes by replacing manual passport stamping procedures with digital records, enabling increased automation, facilitating traveller processing, and supporting a more seamless border-crossing experience. Although the introduction of biometric enrolment requirements may initially increase processing times at border crossing points, solutions such as traveller pre-registration and self-service technologies have the potential to mitigate this impact while preserving the security benefits of the system."* -------------------------------------------------------------------------------------------------------- We thank you once again for taking the time to review the document and provide your constructive feedback. We believe that the revisions introduced have strengthened the manuscript by improving the clarity of the contributions, highlighting the novelty of the proposed solution, providing a explanation of the Entry/Exit System.

# Reviewer report: Reviewer response for version 1

**DOI:** 10.21956/openreseurope.24593.r74218

**Published:** 2026-05-22

**Authors:** Nikolaos Pitropakis

**Affiliations:** 1 Department of Computing and Mathematics, The American College of Greece, Aghia Paraskevi, Greece

The authors of this paper focused on the border crossing and specifically on the issues raised under the new Entry/Exit System (EES) in the European Union. To address delays and improve the passenger experience, they propose instead an application which, according to real-world testing, improved the efficiency of the border crossing process and, of course, the customer experience while keeping the necessary data protection and cybersecurity standards.

 The paper is very timely and, of course, important. However, there are a few issues that the reviewer would like to raise with the authors.

 It would be good to add the contributions of this work in a bullet point format within the introduction section.

 The state-of-the-art section could benefit from a summary table that could highlight the novelties of the proposed solution.

 When it comes to privacy, data protection and cybersecurity explanations, the solutions are listed without proper justification. The authors should focus on the challenges and how they are addressed using the aforementioned solutions.

 When it comes to the registration of the biometrics through the app, more details should be provided about the sensors that can offer the required quality that matches the airport standards.

Is the study design appropriate and does the work have academic merit?

Yes

Is the work clearly and accurately presented and does it cite the current literature?

Yes

If applicable, is the statistical analysis and its interpretation appropriate?

Yes

Are all the source data underlying the results available to ensure full reproducibility?

No source data required

Are the conclusions drawn adequately supported by the results?

Yes

Are sufficient details of methods and analysis provided to allow replication by others?

Partly

Reviewer Expertise:

cybersecurity, cloud computing security, adversarial machine learning

I confirm that I have read this submission and believe that I have an appropriate level of expertise to confirm that it is of an acceptable scientific standard, however I have significant reservations, as outlined above.

# Response

**Published:** 2026-06-24

**Authors:** Joanna Darmos

**Affiliations:** Research and Innovation, European Border and Coastguard- Frontex, Warsaw, Poland

Dear Reviewer, We would like to sincerely thank the reviewer for the positive assessment of our manuscript and for the constructive comments and suggestions. We have carefully considered all remarks and have revised the manuscript accordingly. Below we provide a detailed point-by-point response to each comment.

 ● Comment 1: “It would be good to add the contributions of this work in a bullet point format within the introduction section.”

 Response: We appreciate the reviewer’s constructive suggestion and have revised the manuscript accordingly. A dedicated contribution list has been added at the end of the Introduction section to clearly highlight the scientific and operational contributions of this work. The following bullet points have been included: The key scientific and operational contributions of this work are summarized below:

- Presentation of the first large-scale operational assessment of a mobile application for traveller pre-registration under Entry/Exit System (EES) conditions, involving more than 1,600 travellers in a real airport environment.
- Development and validation of an integrated pre-registration solution combining biometric enrolment, liveness detection, electronic passport verification, and conditions-of-entry questionnaires within a single mobile platform.
- Introduction and assessment of a multi-traveller registration capability supporting families, minors, and organised travel groups.
- Demonstration of measurable operational benefits, including an average reduction of approximately 30 seconds in border processing times and a 25% improvement in efficiency compared with standard EES testing procedures.
- Design and validation of a privacy-preserving and cybersecurity-by-design architecture compliant with European legal and regulatory requirements.
- Provision of practical lessons learned and operational recommendations for future deployment of mobile pre-registration solutions across EU external borders.

● Comment 2: “The state-of-the-art section could benefit from a summary table that could highlight the novelties of the proposed solution.”

 Response: We agree with the reviewer and have added a comparative summary table in the State-of-the-Art section. The table compares existing traveller applications and pilot projects against the proposed QuickBorder solution considering biometric verification, eMRTD chip access, liveness detection, group registration support, operational deployment, integration with border management systems, and compliance considerations. To further emphasize the novelty of our approach, the following paragraph has been added after the comparison table: "While several existing traveller applications focus primarily on travel authorization, customs declarations, or isolated digital identity functionalities, the proposed QuickBorder solution combines biometric enrolment, liveness detection, electronic passport validation, conditions-of-entry questionnaires, and multi-traveller registration within a single operational platform. Unlike previous initiatives that were either limited to pilot demonstrations or specific digital travel credential concepts, QuickBorder was evaluated under real operational conditions at a major international airport and integrated with national border management systems. Furthermore, the solution was designed from the outset following privacy-by-design, cybersecurity-by-design, and regulatory compliance principles, providing a comprehensive framework for future EES-compatible traveller pre-registration systems." **Solution** **Travel Authorization** **Facial Biometrics** **Liveness Detection** **eMRTD Chip Reading** **Group Registration** **Real Operational Testing** **Integration with Border Systems** ArriveCAN ✓ ✗ ✗ ✗ ✗ ✓ Limited ETA Australia ✓ ✓ Partial ✗ ✗ ✓ Limited MPC ✗ ✓ ✓ ✗ ✗ ✓ ✓ DTC1 Pilot ✗ ✓ ✓ ✓ ✗ Pilot Partial KTDI ✗ ✓ ✓ ✓ ✗ Pilot Partial QuickBorder (proposed) ✓/Pre-registration ✓ ✓ ✓ ✓ ✓ (1,600+ travellers) ✓      

 ● Comment 3: “When it comes to privacy, data protection and cybersecurity explanations, the solutions are listed without proper justification. The authors should focus on the challenges and how they are addressed using the aforementioned solutions.”

 Response: We appreciate this observation and have introduced new paragraphs in Sections 6.1 and 6.3 to focus on the challenges addressed and the rationale behind the implemented measures rather than simply listing the controls. The following text has been incorporated into the Privacy and Data Protection subsection: "The collection and processing of biometric and biographic information within a mobile environment introduces significant privacy and data protection challenges. The App4EES solution was designed to operate in real operational environments using real traveler data, making the protection of individuals' personal information a primary design objective. The implemented privacy-by-design and privacy-by-default approach addresses risks associated with excessive data collection, unauthorized access, unlawful processing, and lack of transparency. Data minimization mechanisms ensure that only information strictly necessary for the pilot objectives is collected. Explicit informed consent, transparent privacy notices, clearly defined retention periods, and mechanisms allowing travelers to revoke consent and request deletion of their data ensure compliance with the principles established by the General Data Protection Regulation (GDPR). In addition, a dedicated Data Protection Impact Assessment (DPIA) was conducted to identify, assess, and mitigate risks associated with the processing of biometric information, ensuring full accountability, transparency, and alignment with European legal and regulatory requirements. Furthermore, despite the fact that the AI Act was not yet applicable during the execution of the pilot, the DPIA incorporated an assessment of risks and mitigation measures aligned with the provisions under discussion in the draft AI Act. This proactive approach facilitated early alignment with anticipated European requirements for AI systems involving biometric processing and strengthened the overall accountability and future readiness of the solution." The following text has been incorporated into the Cybersecurity subsection: "The use of mobile devices for biometric enrolment introduces a broad threat landscape that includes unauthorised access attempts, interception of sensitive information during transmission, credential theft, presentation attacks against facial recognition systems, malware infections, device compromise through rooting or jailbreaking, backend service abuse, and denial-of-service attacks. To address these challenges, the system was designed following a cybersecurity-by-design approach. Sensitive operations involving biometric verification and travel document validation are executed within trusted backend environments rather than on user devices. Strong cryptographic protections safeguard data both at rest and in transit, while authentication and authorisation controls protect access to services. Additional measures include anti-tampering controls, device integrity verification, API throttling, secure communications, liveness detection mechanisms to mitigate presentation attacks, and continuous monitoring capabilities. The adoption of the OWASP Mobile Application Security Verification Standard (MASVS) provides a structured framework to ensure resilience against known mobile application threats. These measures not only protect the system against cybersecurity risks but also contribute to maintaining the integrity, reliability, and legal trustworthiness of the overall border management process."

 ● Comment 4: “When it comes to the registration of the biometrics through the app, more details should be provided about the sensors that can offer the required quality that matches the airport standards.”

 Response: We thank the reviewer for highlighting this important aspect. Additional clarification has been included in Section 4.2 (Frontend User Interface) regarding the biometric acquisition process and sensor requirements. The following paragraph has been added: "Facial biometric enrolment is performed using the traveller's native smartphone camera without requiring any dedicated external sensor. Modern smartphone cameras provide image quality that is generally sufficient for biometric verification purposes, particularly when combined with the implemented quality assessment and liveness detection mechanisms. The objective of the mobile enrolment process is to perform remote identity verification and facilitate pre-registration before arrival at the border. However, if the image acquired through the mobile device does not meet the quality thresholds required for enrolment in operational EES records, a compliant facial image can still be acquired at the border crossing point using border-control equipment. Consequently, the mobile application complements rather than replaces existing border biometric acquisition procedures and provides operational flexibility while maintaining compliance with applicable biometric quality requirements."

 We thank you once again for taking the time to review and provide constructive feedback. We believe that the revisions introduced have strengthened the manuscript by improving the clarity of the contributions, highlighting the novelty of the proposed solution, providing a stronger justification for privacy, data protection, and cybersecurity measures, and clarifying the biometric acquisition process.
